# Inter-protein residue covariation information unravels physically interacting protein dimers

**DOI:** 10.1186/s12859-020-03930-7

**Published:** 2020-12-17

**Authors:** Sara Salmanian, Hamid Pezeshk, Mehdi Sadeghi

**Affiliations:** 1 Department of Bioinformatics, Institute of Biochemistry and Biophysics, University of Tehran, Tehran, Iran; 2 School of Mathematics, Statistics and Computer Science, College of Science, University of Tehran, Tehran, Iran; 3 Present Address: Department of Mathematics and Statistics, Concordia University, Montreal, Canada; 4 School of Biological Sciences, Institute for Research in Fundamental Sciences, Tehran, Iran; 5 National Institute of Genetic Engineering and Biotechnology, Tehran, Iran

**Keywords:** Protein–protein interaction, Physical interaction, Sequence-based prediction, Coevolution, Surface accessibility, Mutual information

## Abstract

**Background:**

Predicting physical interaction between proteins is one of the greatest challenges in computational biology. There are considerable various protein interactions and a huge number of protein sequences and synthetic peptides with unknown interacting counterparts. Most of co-evolutionary methods discover a combination of physical interplays and functional associations. However, there are only a handful of approaches which specifically infer physical interactions. Hybrid co-evolutionary methods exploit inter-protein residue coevolution to unravel specific physical interacting proteins. In this study, we introduce a hybrid co-evolutionary-based approach to predict physical interplays between pairs of protein families, starting from protein sequences only.

**Results:**

In the present analysis, pairs of multiple sequence alignments are constructed for each dimer and the covariation between residues in those pairs are calculated by CCMpred (Contacts from Correlated Mutations predicted) and three mutual information based approaches for ten accessible surface area threshold groups. Then, whole residue couplings between proteins of each dimer are unified into a single Frobenius norm value. Norms of residue contact matrices of all dimers in different accessible surface area thresholds are fed into support vector machine as single or multiple feature models. The results of training the classifiers by single features show no apparent different accuracies in distinct methods for different accessible surface area thresholds. Nevertheless, mutual information product and context likelihood of relatedness procedures may roughly have an overall higher and lower performances than other two methods for different accessible surface area cut-offs, respectively. The results also demonstrate that training support vector machine with multiple norm features for several accessible surface area thresholds leads to a considerable improvement of prediction performance. In this context, CCMpred roughly achieves an overall better performance than mutual information based approaches. The best accuracy, sensitivity, specificity, precision and negative predictive value for that method are 0.98, 1, 0.962, 0.96, and 0.962, respectively.

**Conclusions:**

In this paper, by feeding norm values of protein dimers into support vector machines in different accessible surface area thresholds, we demonstrate that even small number of proteins in pairs of multiple alignments could allow one to accurately discriminate between positive and negative dimers.

## Background

Proteins are key functional molecules playing critical roles inside cells. These important biomolecules accomplish their roles using inter-molecular interactions [1, 2]. Protein–protein interactions (PPI) are involved in numerous cellular processes [3, 4]. Most proteins accomplish their tasks through physical interactions. Prediction of those physical interplays is a grand challenge in computational biology [5]. There are considerable various protein interactions and myriads of protein sequences and synthetic peptides with unknown interacting counterparts waiting to unveil. Therefore, developing new approaches for accurate prediction of physical interactions among proteins directly from primary amino-acid sequence would be a breakthrough in the field of bioinformatics.

Protein coevolution occurs to prevent disrupting a critical interaction. In this case, mutations in key interfacial residues in one of the interacting pairs of proteins enforce compensatory mutations in the other one. Therefore, two interacting proteins coevolve through interdependent changes at their interaction interface [6, 7]. This residue coevolution can be exploited to decipher specific physical interacting proteins. Some of sequence-based approaches to infer PPI use coevolution at amino acid level but others apply it at protein level mostly using principles of molecular phylogenetic [6].

Coevolution at the level of protein sequence potentially discovers both physical and functional interactions [8–13]. A pair of proteins tend to interact if their correspondent protein family coevolve [14, 15] and possess cognate phylogenetic tree with similar distance matrices [8, 10, 16–18]. A dozen of "Mirror-tree"-based studies are focused on deciphering protein-level interaction. Those studies measure the similarity of distance matrices of two phylogenetic trees by correlation coefficient. All of those methods predict physical interaction or functional associations between two protein families [8–13] or inside pairs of Multiple Sequence Alignments (MSAs) [14, 17, 19, 20].

So far, residue level coevolution is exploited to decipher residue contact maps inter and intra proteins. Physical interactions between pairs of protein sequences are indeed local phenomena and occur in specific interfacial residue components [6]. Accordingly, groups of co-evolutionary physical PPI prediction methods (hybrid methods) are extended in which inter-protein residue coevolution is employed to predict the interaction between protein molecules in higher scales. Given that PPI inference through hybrid methods is performed by prior inter-protein residue contact map prediction, those approaches are applied to infer specific physical PPI.

In literature, the problem of hybrid PPI inference approaches has thus far been presented in two different concepts [21, 22]. Some of those approaches explore the interaction between specific protein partners inside a paired MSA [23–30] whereas a handful of others assess the possibility of interaction between protein families [31–33]. Among the former approaches, Bitbol (2018) addressed paralogous problem within pairs of protein families. She applied Iterative Pairing Algorithm (IPA) to maximize the final co-evolutionary signal and predicted the best possible matchings between protein partners in each paired MSA [24]. The second group of approaches are desired for deciphering the physical couplings between pairs of protein families. The growing need to build accurate datasets and computational costs of building hundreds of paired MSAs for hundreds of putative pairs of proteins and employing residue coevolution on those datasets has limited those studies. Nevertheless, a few studies have thus far focused on this methodology. Our method is among this group. In 2002, Pazos et al. proposed a basic idea, in-silico two hybrid (i2h), that correlation between pairs of residues in paired MSAs of proteins is sufficient for reconstruction of protein interaction networks. Application of that system on various test sets revealed that i2h has a good capacity to discern between true and false interactions [31]. Feinauer et al. (2016) applied plmDCA on paired MSAs of simulated and biological pairs of proteins. They tested their co-evolutionary analysis on ribosomal and tryptophan operon proteins and indicated that residue coevolution is strong enough to discriminate interacting protein families from non-interacting family pairs [32]. In a recent study, Cong et al. (2019) proposed a hybrid PPI prediction method in which they performed sets of residue coevolution screenings on several protein benchmark datasets and predicted the possible physical interactions among proteins. They indicated that their screen outperforms several experimental procedures [33].

Local and global residue co-evolutionary methods have thus far been used to unravel physical PPI. In local residue coevolution approaches including Mutual Information (MI) [34, 35], McBASC [36] and so forth, each pairs of residues are considered independent of other residues whereas in global approaches as CCMpred [37] and so forth [38–44], the correlation between each residue pair is taken into account.

In the present survey, a hybrid method is applied in which three MI-based methods and a single representative global approach, CCMpred, are employed for residue coevolution analysis for further physical PPI prediction. A set of Accessible Surface Area (ASA) thresholds are considered and residues exceeding those cut-offs are assumed as exposed residues. Inter-protein residue contact matrices are acquired in different ASA groups for each putative pair of proteins for CCMpred and MI-based approaches. There are various magnitudes of signals in each residue contact matrix most of which are indirect noisy signals. In order to attenuate the impact of those signals, we take advantage of a novel innovative application of "Frobenius norm" concept. Whole elements of residue coevolution matrix of each interacting pair are summarized into a single "norm" value. The aim is to reduce the effect of noisy signals by considering power of two of the elements of the matrix [see expression (9)]. This approach both reduces the effect of noises and amplifies the power of direct residue interactions.

The present study indicated that the whole entries of residue coevolution matrices are strong enough to discriminate interacting protein families from non-interacting family pairs.

## Results

### Overview

**Fig. 1:**
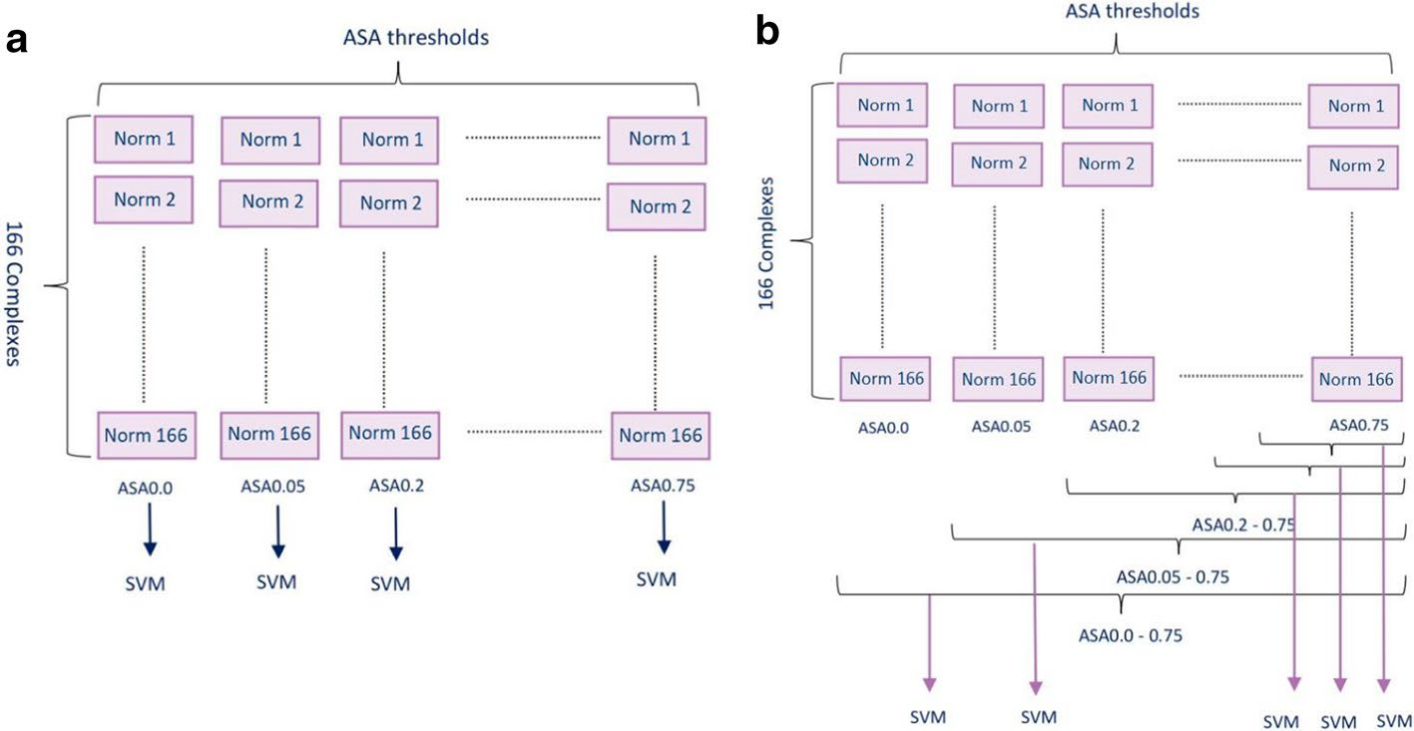
Contribution of features to SVM classifications. **a** For each single ASA threshold, SVM is trained by norm values of residue contact matrices as a single input feature for further prediction. **b** SVM models are developed for multiple groups norm values in different sets of ASA thresholds to predict the possible interaction in each heterodimeric pair

In this study, a set of gold standard positive and control negative heterodimers are examined to infer physical PPI only based on sequence-based co-evolutionary information. Pairs of MSA families are constructed for each dimer and the covariation between residues in those pairs are calculated by CCMpred and three MI-based approaches for ten ASA threshold groups. Frobenius norms of residue contact maps between interacting pairs of proteins are calculated for all dimers in those cut-offs (see Additional file 1). At the next step, several SVM classifications are developed to distinguish between positive and negative heterodimeric protein couples. The input features of SVM models are Frobenius norms of covariation matrices. Norms of inter-protein residue contact matrices of all dimers in different ASA thresholds are fed into SVM as single (Fig. 1a) or multiple feature (Fig. 1b) models. Finally, the prediction performance of each model is evaluated using accuracy, sensitivity, specificity, precision, NPV, FPR, FNR and BM.

### Datasets

The final remaining numbers of samples in our datasets are 166 couples of proteins in which 83 equal numbers of dimers exist in both positive and negative datasets.

More than 75% of whole datasets contain less than $\sim 450$ sequences. Distributions of the numbers of sequences in MSA datasets are available in Additional file 2: Fig. S1.

Except a handful of deeper MSAs with $N/L$ ratio of more than 5, nearly all alignments are shallow. The ratios for half and three-fourth of all putative pairs of protein families are less than ∼1 and ∼1.7, respectively. Distributions of $N/L$ ratio in MSA datasets are shown by Additional file 2: Fig. S2.

In average, 96% of proteins in our datasets were prokaryotic and other remaining 4% were for unknown organisms.

To know whether the final sequences in each MSA are orthologous to query protein sequence, the identity of GO terms (molecular function, biological process and cellular component) of those protein sequences is compared and a final relative frequency of identical terms in each MSA is measured. Except a few outliers, that index for all MSAs fall within the range of > 0.93 to > 0.995 at the median of ∼0.97, for all three GO terms which approves qualified orthologous selection. Since ∼0.34 of each MSA are averagely related to unknown proteins, lower relative frequencies of GO terms are in part due to unknown sequences within protein families. Boxplots of the identity of GO terms among proteins in our MSA families is displayed in Additional file 2: Fig. S3.

We speculate that pairs of interacting proteins are involved in the same biological processes and are located inside equivalent cellular components and therefore those partners in each pair of protein families could be matched by the identity of aforementioned GO terms. The GO function of those protein partners could be identical, similar or complementary. In this study, we also assessed the identity of GO terms between protein counterparts in each pair of MSAs and reported the relative frequency of identical pairs (see Additional file 2: Fig. S4). In this case, the median levels of all three GO terms are approximately 0.96. However, unlike two other GO terms, GO function relative frequencies ranges widely, from ∼0.65 to 1 excluding several outliers.

We also compare covariation value distribution of expose residues with buried ones for different ASA thresholds. Those distributions show an obvious distinction among different cut-offs in CCMpred approach between exposed residues but no apparent difference is observed in three MI-based methods (illustrated in Additional file 2: Fig. S5).

### SVM predictions

#### Single feature models

**Fig. 2:**
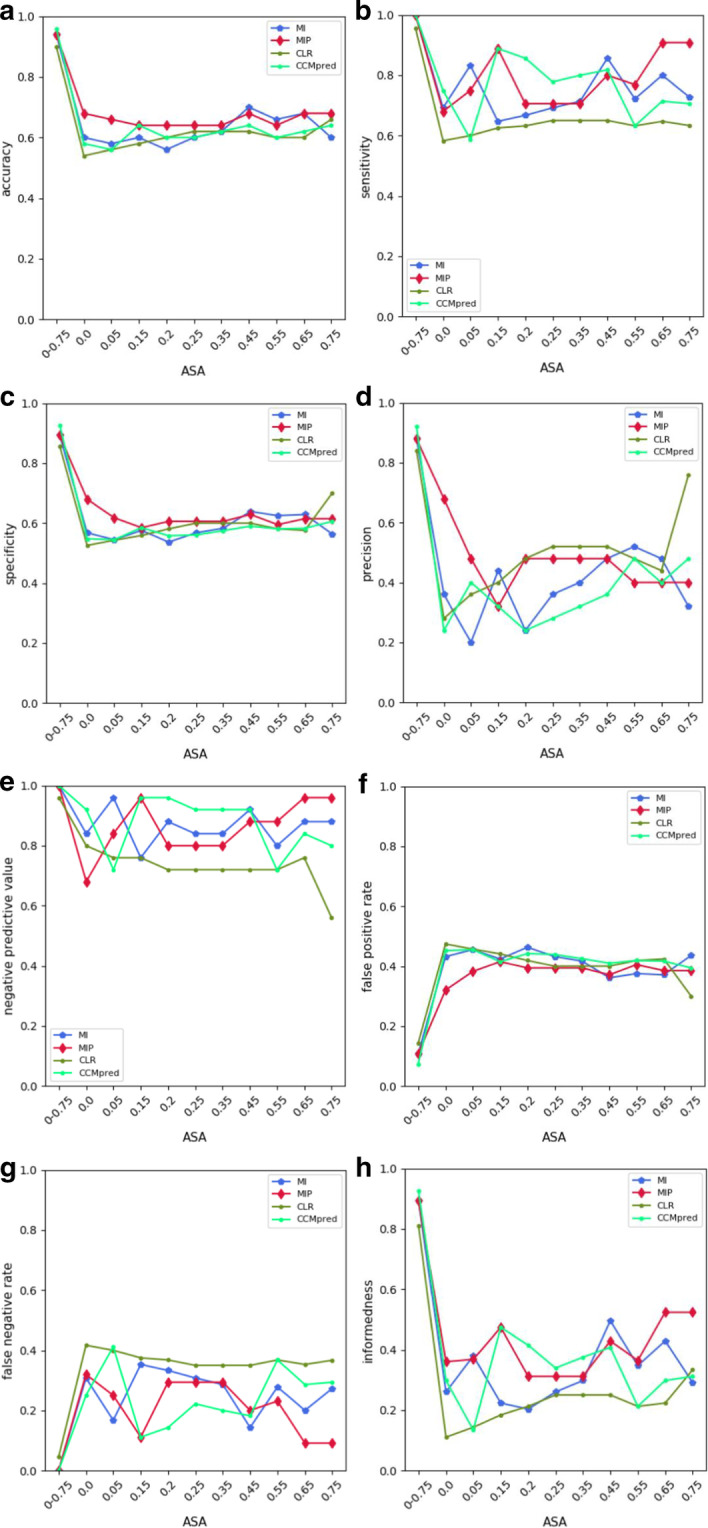
Prediction of evaluation metrics for SVM models developed by single norm features in separate ASA thresholds in comparison with all norm features at all ASA thresholds in CCMpred and three MI-based methods. Accuracy, sensitivity, specificity, precision, NPV, FPR, FNR and Informedness (BM) are, respectively, denoted by **a**, **b**, **c**, **d**, **e**, **f**, **g**, **h**. *Note:* ASA 0–0.75 is a set of norm values for all ASA thresholds from ASA0.0 to ASA0.75

The input feature of each SVM model is norm values for each ASA threshold (see Fig. 1a and Additional file 1). SVM models are separately developed for ten ASA thresholds and the prediction results are finally obtained for each interacting proteins of each dimer in test set. The results are shown in Fig. 2 and Additional file 3. The results of single feature models (single norm value for each separate ASA threshold) are compared with all feature model (norm values for all ASA thresholds from 0.0 to 0.75) and presented in those figures and tables. As shown by Fig. 2, modelling SVM using all ASA groups of norm values (ASA0-075) represents much better accuracy, sensitivity, NPV, FPR, FNR and BM results than single ASA thresholds, thereby collectively providing much superior performance than single input features.

In all four approaches especially in MIp and CLR, increasing ASA shows an overall constant trend for accuracy, specificity and FPR results. The results of those metrics are close to each other, albeit slightly higher in MIp, for all cut-offs.

The highest single ASA sensitivity, NPV and BM results are related to ASA 0.65 and ASA 0.75 in MIp procedure. In the case of CLR method, those indices have an overall constant trend for all ASA threshold groups, roughly the lowest performance in comparison with other three.

In comparison with other approaches, the highest numbers of TP and FN, and the lowest numbers of FP and TN are obtained for ASA 0.75 in CLR method which results in the highest precision and specificity and the lowest NPV and FPR. This threshold collectively behaves different from other CLR cut-offs. The lowest sensitivity, accuracy and BM is also obtained in the case of ASA 0.0 in CLR approach.

Additional file 2: Figures S6 and S7 respectively represent distributions and box plots of norm values in CCMpred and MI-based methods in two datasets (positive and negative control) for different single ASA thresholds. As denoted by those figures, both CCMpred and MIp methods better distinguish norm values of positive and negative putative interacting pairs of proteins in comparison with the other two approaches. A Mann–Whitney U test was performed with a significance level of 5% or lower to compare norm values of positive and negative datasets in different ASA thresholds. The results indicated that those datasets are significantly different in all ASA thresholds in CCMpred and MIp approaches (*p* value < 0.05). The difference between averages of norm values of positive and negative datasets are only significantly different at ASA = 0.65 in MI method, and ASA = 0.45 to ASA = 0.65 in CLR approach (Additional file 2: Fig. S7).

#### Multiple feature models

**Fig. 3:**
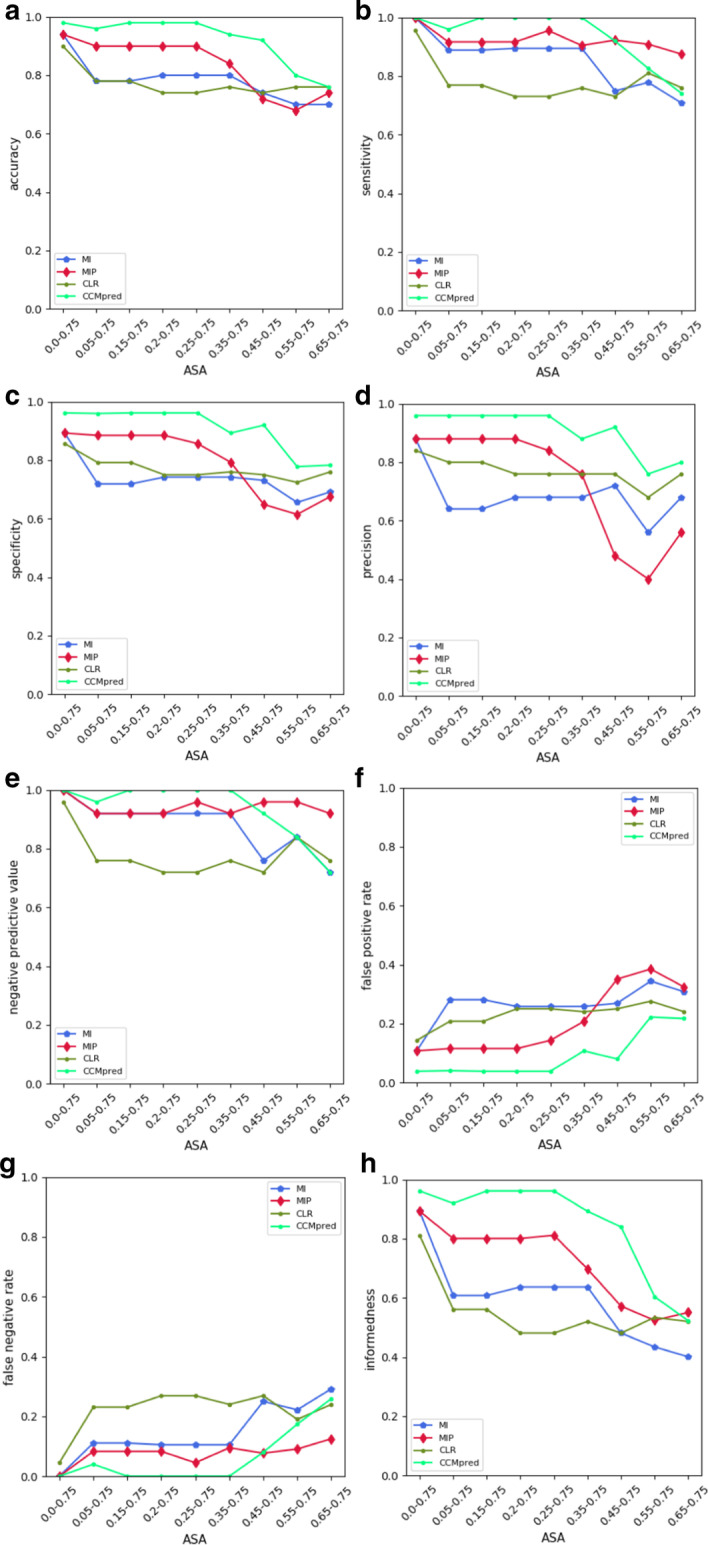
Prediction evaluation results for SVM models developed by multiple norm sets in series of ASA threshold groups in CCMpred and three distinct MI-based approaches. Multiple norm sets of ASA thresholds are employed to train SVM in 2 to 10 feature groups. Accuracy, sensitivity, specificity, precision, NPV, FPR, FNR and Informedness are, respectively, denoted by **a**, **b**, **c**, **d**, **e**, **f**, **g**, **h**. *Note:* 0.0–0.75 is a set of norm values for all ASA thresholds, ASA0.0 to ASA0.75. All other sets of ASA thresholds have similar symbols

We develop SVM models using multiple feature sets of norm values for ASA threshold groups from 0.0 to 0.75 (see Fig. 1b). Multiple norm sets of ASA thresholds are applied for training SVM models in groups of 2 to 10 features. Figure 3 and Additional file 4 illustrate a comparison between prediction results for different sets of multiple features in multiple ASA threshold groups. Increasing the numbers of input feature sets improves prediction results of SVM models.

As shown by Fig. 3, CCMpred has an overall higher accuracy, specificity, precision, FPR and BM in comparison with the other three approaches. Although more accurately discriminates positive and negative dimers than other three approaches, CCMpred method undergoes downtrend in four feature groups (0.35–0.75, 0.45–0.75, 0.55–0.75, 0.65–0.75) due to uptrend in the number of FPs. Meanwhile, CCMpred represents the best sensitivity, NPV and FNR for all sets of ASA thresholds other than three feature groups (0.45–0.75, 0.55–0.75, 0.65–0.75), where it undergoes a sustained downtrend reaching the worst value for ASA 0.65–0.75. Since BM depends on both sensitivity and specificity, the concomitant reduction in the numbers of FP and FN for three aforementioned feature groups leads to an even greater decline in BM index in comparison with constructing indices.

Collectively, MIp is the second qualified approach which represents an overall higher sensitivity, NPV and BM in comparison with CLR and MI. Persistent numbers of TN and FN in all sets of cut-off groups results in a roughly constant trend for sensitivity and NPV values in MIp which also lead to higher values in three feature groups (0.45–0.75, 0.55–0.75, 0.65–0.75) than other three approaches. In MIp method, a sudden downfall and raise of the numbers of TP and FP in four feature groups (0.35–0.75, 0.45–0.75, 0.55–0.75, 0.65–0.75), respectively, leads to a sudden decline of accuracy, specificity and precision indices. In that method, threshold groups of ASA 0.45–0.75 and ASA 0.55–0.75 achieve a coincident worst numbers of TP and FP, and thereby the lowest of mentioned indices in comparison with all other approaches.

Altogether CCMpred, MIp, MI and CLR approaches, respectively, represent the best accuracy, sensitivity, NPV and BM in all sets of threshold groups other than three sets (0.45–0.75, 0.55–0.75, 0.65–0.75). Therefore, CCMpred and CLR methods are the most and the least accurate, sensitive, informative and negative value predictor approaches in those feature sets, respectively. On the other hand, CLR achieves an overall higher specificity and precision than MI. Therefore, all the above mentioned rules are met excluding the order of CLR and MI for specificity and precision metrics.

## Discussion

In this study, we introduced a hybrid co-evolutionary-based method to predict physical PPI between pairs of protein families, starting from protein sequences only. Here, physically interacting proteins exhibit more strongly co-evolutionary signals (norm values) than negative group. We hybridized inter-protein residue-level coevolution with protein scale one by unification of whole residue covariation map between proteins of each dimer into a single norm value. Generally, hybrid methods more specifically predict physical PPI than mere protein-level co-evolutionary approaches such as mirror-tree [8–13], on account of considering whole interfacial residue interaction information as building blocks of proteins and contributing factors in physical PPI at lower scales [31–33]. By training SVM with norm values of positive and negative dimers in either distinct or several ASA thresholds, we demonstrated that even small number of orthologous proteins in pairs of MSAs could allow one to accurately discriminate between positive and negative dimers.

In the present work, several filtrations finally gave rise to the construction of shallow MSAs distinguished enough for covariation analysis. The advantages of the current study include accurate prediction of physical PPI in small alignments and lower computational costs in the context of training with single features especially in higher ASA cut-offs. Nevertheless, PPI prediction in those alignments considerably improves at the expense of higher computational costs in the case of training SVM by several norm features in multiple ASA thresholds.

A diverse group of protein complexes is selected in this study in which dimeric pairs of interactions are picked. Although assessing physical PPI on whole interactome is desired, the present experiment is not employed on proteome network scale on account of several limitations. Groups of designated dimers were discarded from our experiment due to limitations on the length of each dimer, the least acceptable number of sequences in each pair of protein families, different filtration steps for dataset construction, outlier removal, and so forth. Meanwhile, although our work has lower computational cost in calculation of inter-protein residue covariation for each pair of protein families, dataset generation steps from sequences to the ten final pairs of ER-MSAs for ten ASA thresholds and calculation of ten covariation matrices for all those pairs are extremely time-consuming for each dimer. In Cong et al. work [33], a sequential covariation screening is executed, where MI between all proteome-wide combinations of proteins in two prokaryotic species is measured at first step followed by a DCA and GREMLIN calculations on selected pairs of proteins, whereas in our analysis CCMpred and three MI-based covariations are measured and compared for each dimer in several pairs of ER-MSAs in parallel which makes proteome-wide analysis beyond the scope of current work.

In this study, GO matching strategy of orthologous sequences to query in each MSA is applied. Altenhoff et al. (2012) tested the identity of GO term annotations among orthologous sequences to assess if they have similar functions [45]. We also investigated the identity of GO terms between the different sequences in the MSAs and their query protein, based on their findings and "Orthologue Conjecture" which assert that orthologous sequences are more functionally similar than paralogous ones [45, 46]. The results showed that except a handful of alignments, all three GO terms including molecular function, biological process and cellular component are considerably similar among proteins of each MSA. Actually, in this study we selected orthologous sequences and tried to remove paralogous proteins. Cong et al. also selected orthologous sequences but by RBH procedure [33]. On the contrary, Feinauer et al. (2016) constructed datasets composed of both paralogous and orthologous sequences [32]. Another matching strategy applied in our work was GO matching between pairs of proteins in pairs of MSAs to acknowledge if pairs of plausible orthologues are coupled and to ensure that erroneously retained paralogs do not match with orthologues. Interacting pairs in each organism should reside on the identical cellular component, take part in the same biological process [47] and often possess complementary or sometimes the same function [48]. Our results indicated that the identity of molecular function between protein partners is lower than two other annotations consisting of wide tolerance due to the fact that the mentioned property is sometimes similar but not identical. In the present work, only orthologous sequences are retained and functionally relevance of those partners in pairs of protein families is verified. That verification could also be promising in providing new insights on matching paralogous proteins between pairs of alignments. In that context, paralogous pairs with identical GO components and biological processes and also similar or complementary GO functions could be simply matched. Therefore, paralogous problem could be readily addressed with less computational costs.

As previously mentioned, the first step after ER-MSA generation is residue coevolution measurement in order to perform a hybrid physical PPI inference method. There are a wide variety of approaches to unravel residue covariation between pairs of proteins. Those approaches include simple local methods as MI [34, 35], McBASC [36], and so forth, and global approaches as CCMpred [37] and others [38–44]. Performing some global covariation methods like PSICOV [44] and GREMLIN [49] entail deep alignments including thousands of sequences, infeasible on some of our shallow MSAs. Accordingly, we infer inter-protein residue coevolution by taking advantage of fast and simple MI-based methods as they could make a fair prediction on small alignments [50].

Inferring inter-protein residue coevolution between interacting pairs of each dimer results in construction of a matrix which contains whole residue coupling information. Residue pairs having strong covariation signals usually interact firmly and are spatially proximal. For that reason, strong signals are often the consequence of direct residue contacts. Most elements of the interaction matrix are weak noisy signals which are the result of indirect residue associations. Frobenius norm value describes and summarizes matrix size and whole matrix values into a single quantity and de-noises matrix entries and thereby more purifies direct residue interactions from background noises.

Based on the previously tested assumption that PPI is more related to surface accessible residues than buried amino acids at the protein core [51–53], we considered surface area as a feature for training SVM. A comparison between covariation score distribution of buried and exposed residues for different ASA thresholds showed that CCMpred possibly differentiates those scores while it appears that no differentiation is observed in MI-based methods. The reason seems to be on account of the fact that covariation by global methods like CCMpred inherently disentangle direct residue interactions from indirect ones, but MI method would probably discriminate those two interactions after employing Frobenius norm by attenuating noisy and intensifying direct signals. Therefore, it sounds that direct covariation signals before applying Frobenius norm are not strong enough to make a discrimination between two buried and two exposed residues in MI. The difference between differentiation power of CCMpred and MI could also be interpreted as more influence of buried residue elimination on MI methods.

MIp and CCMpred methods better discriminate positive and negative datasets than CLR and MI. Indeed, MIp removes the background noise imposed by all other residue couplings and also eliminates the influence of phylogeny or entropy from each residue contact. Therefore, that method more purifies signals related to more strongly coevolving positions and direct residue interactions between couples of protein families [54]. CCMpred as a representative DCA-based method also discriminates direct residue couplings from merely correlated indirect contacts [37]. Removing the noise and entropy by MIp and indirect signals by CCMpred, results in boosting more strongly co-evolutionary signals and better discrimination of norm values between positive and negative datasets than CLR and MI approaches. Different sources of noise are available in MI and CLR methods which restrict significant discrimination of norm values between datasets to specific ASA thresholds.

The results of training the classifiers by single features i.e., norm values of residue contact matrices, showed no apparent different accuracies in distinct methods for different ASA thresholds. Nevertheless, comparison of covariation approaches by other evaluation metrics demonstrated that MIp may roughly have an overall better performance than CLR method. It seems that optimal ASA threshold is varied in different kinds of proteins. To meet different optimality properties of various dimers, we trained SVM with multiple norm features for several ASA thresholds which led to a considerable improvement of prediction performance. The results of training with multiple features demonstrated that increasing the number of features results in an overall uptrend in the accuracy and BM. Meanwhile, CCMpred roughly performs better than other approaches in the case of training with multiple feature sets. Here, the best accuracy, sensitivity, specificity, precision and NPV for that method are 0.98, 1, 0.962, 0.96, and 0.962, respectively. In contrast to our PPI prediction procedure, the best reported precision by Cong et al. for Mycobacterium tuberculosis was 0.83 [33]. Additionally, Feinauer et al. reported the sensitivity value of 0.11 and 0.7 for whole and top10 interactions, respectively [32]. These findings indicated that our procedure better predicts physical PPI than two other approaches. However, accurate comparison between several methods require the identical input dimers and similar outputs, but both of those criteria and methodologies are different between our method and two others. Our better prediction performance could either be due to our different methodology or our small sample size. Unlike other studies, our results are based on whole but not merely top covariation values. Actually, no threshold is delineated for those values in our analysis but a binary yes–no prediction. However, all of three analysis indicate that residue coevolution could be exploited to accurately discriminate between positive and negative dimers in order to infer physical PPI [32, 33].

There are several limitations in the present study. Although shallow alignments increase the speed of covariation calculations, executing different filtrations for dataset generation is time consuming. Additionally, our analysis is constrained by the number of dimers. Less stringent filtration criteria could be performed both to prevent elimination of a large number of homologous sequences and final preliminary dimers to get deeper alignments for being able to test more residue co-evolutionary methods on the datasets. Meanwhile, deep alignments could be obtained by addressing paralogous problem. The results should be generalized to higher proteome scales and finally a number of unknown PPIs could become candidates as plausible physical PPI for further biological experiments. Meanwhile, eukaryotic protein dimers could be applied in future to see whether our method could also make an acceptable prediction in the context of those organisms. However, since there are usually a large number of paralogs in eukaryotes, addressing that problem is computationally a hard problem. Protein dimers with a huge number of conserved residue pairs are not qualified for co-evolutionary analysis like ours but a corpus of residue columns with co-mutating patterns are required.

Since our filtration pipeline for dataset generation can find and select high percentage of orthologues, this screening procedure could also be promising for orthologous selection in future. Meanwhile, as mentioned earlier, GO term matching procedure would appear to be a promising way for coupling paralogous proteins in pairs of alignments. In this case, instead of coupling by simple GO term identities, matching GO term semantic similarities could be considered in future. But of course other efficient paralogous matching methods including IPA [23, 24] and Ouroboros [55] exist which could also be taken into account, even though constrained by computational cost. Prediction of physical PPI based on residue coevolution is an ongoing field. Although there are a handful of methods including ours to address the problem, but accurate methods for the prediction of proteome-wide physical interactome especially for eukaryotic proteins are still demanding.

## Conclusions

In this paper, a hybrid co-evolutionary approach is introduced. The goal of this study is to exploit inter-protein residue coevolution to accurately decipher physical interaction at higher scales between pairs of protein families in sets of protein dimers. The covariation between residues in those dimers are calculated by CCMpred and three MI-based approaches for ten ASA threshold groups. Here, whole residue contact map between proteins of each dimer are summarized into a single norm value. We train SVM with norm values of residue contact matrices of all dimers at different ASA thresholds as single or multiple feature models. The results demonstrate that training SVM with multiple norm features leads to a considerable improvement of prediction performance, but classifiers trained by single features show no apparent different accuracies in distinct methods at different ASA thresholds. Nevertheless, in the case of single feature models, MIp roughly achieves an overall better performance than three other methods. The findings also indicate that an overall performance of CCMpred is higher than other three approaches in the context of multiple feature SVM models.

Finally, we demonstrate that even small number of proteins in pairs of MSAs could allow to accurately discriminate between positive and negative dimers. The results should be generalized to higher proteome scales. Prediction of physical PPI based on residue coevolution is an ongoing field and developing accurate methods for the prediction of proteome-wide physical interactome especially for eukaryotic proteins are still demanding.

## Methods

The goal of this study is employing interaction at residue level for accurately inferring physical PPI at protein level. To achieve this goal, we perform several steps consecutively including dataset construction, calculation and unification of whole inter-protein residue coevolution and discrimination of positive and negative heterodimers.

**Fig. 4:**
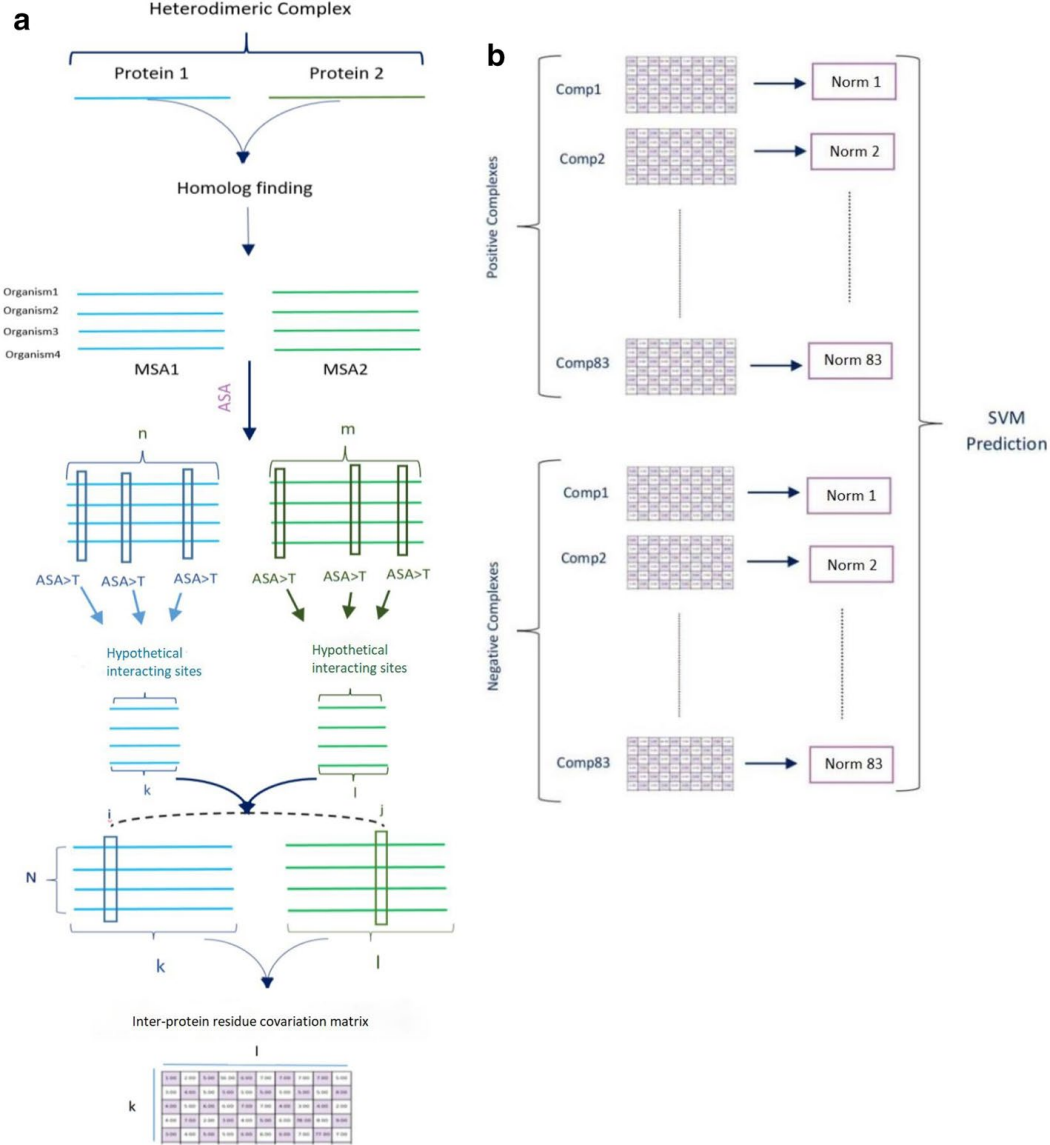
Overview of our workflow. **a** Sequence of each protein has been searched against reference proteome for finding homologous sequences. MSA is built for both protein chains of each pair. Then, ASA thresholds are determined to select exposed residues. Finally, inter-protein residue interaction matrix is built for each pair of interacting site. **b** Each coevolution matrix is unified into a single "norm" quantity. Norm values are calculated for 166 dimers (83 positive and 83 negative putative pairs of proteins) in each ASA threshold which are fed into SVM as input features for further prediction. comp: protein complex (Heterodimers in our analysis)

The workflow is illustrated in Fig. 4.

### Dataset construction

Couples of non-identical interacting proteins (heterodimers reported in [42, 49]) are selected from Protein Data Bank (PDB) verified complexes to construct a positive gold standard dataset. Furthermore, pairs of non-identical proteins are randomly picked from different positive complexes to generate a negative control dataset.

We defined the length of each heterodimer (L) as the square root of the product of l_1_ and l_2_, where l_1_ is the length of protein1 and l_2_ is the length of protein2. Positive and negative heterodimers with length > 550 and < 90 are removed from the analysis. We discarded some of differently distributed negative heterodimers in order to equally distribute the length of positive and negative heterodimers. Length distributions of positive and negative dimers are compared in Additional file 2: Fig. S8.

Sequences of interacting pairs of proteins are downloaded from UniProtKB [56]. Then PSI-search SOAP service is run for 3 iterations against reference proteome to find homologous sequences for each query protein sequence. In each iterative step, a group of eligible homologous protein sequences are selected by applying a set of filtration criteria (see Table 1) to build PSSM matrix which are further searched against reference proteome [57]. Those criteria include sequence length, identity, bit score, e-value and overlap length (see Additional file 2: Section 1.2 for more details).

**Table 1:** Filtration criteria to sieve homologous sequences in each PSI-search iteration

Homolog filtration criteria	
Sequence length	At least 80% of the query length but 78% in special cases
Sequence identity	Sequence identity of at least 40% [70] but 25%, 30% or 35% in special cases
Bit score	Bit score threshold of 0.5 $\times$ monomer sequence length [42] and 0.45 $\times$ monomer sequence length in special cases
E-value	E-value smaller than 0.0001
Overlap length	Overlap length threshold of at least 90% but 70%, 80% or 85% in special cases

For addressing paralogous problem, our strategy is selecting orthologous sequences among homologous proteins while still eliminating paralogous ones. There are four criteria for designating orthologous sequences [58] that often one of them are applied for tackling paralogous problem. Those criteria include "sequence similarity" like Reciprocal Best Hit (RBH) procedure in [33], "synteny" like genomic co-localization in [32, 42, 49], "phylogenetic tree matching" like [59], and "functional complementarity" [58].

We applied both sequence and functional similarity criteria in order to select orthologous and eliminate paralogous sequences. Detailed pipeline of orthologous selection is demonstrated in Additional file 2: Section 1.1 and Fig. S9. Orthologous protein sequences are acquired for each query protein of each heterodimer. Those sequences consisting of common species between two protein families of each dimer are retained and arranged in the same organism order for further alignment while others are removed.

Homologous sequences already arranged in the specific order are aligned using Mafft G-ins-i [60–62] version 7.312 (mafft—maxiterate 1000—globalpair—clustalout). After constructing first MSA, outlier sequences (possibly paralogous sequences) and their counterparts from other protein family are removed (see Additional file 2: Section 1.1 and Section 1.3 and Fig. S10 respectively for outlier definition, detection algorithm and examples of outliers). There are large numbers of outliers in some alignments and these outliers can also deviate MSA from ideal case. Hence, multiple alignment is done again with remaining sequences. At the second multiple alignment, those columns of MSA in which query sequence contains gaps, are removed.

To ensure that the final MSA datasets are made of orthologous sequences, the relative frequency of one or more common identical GO term annotations between proteins in each MSA and the query protein is measured. Furthermore, to certify that pairs of putative orthologous proteins within each pair of MSAs are correctly matched, we obtain the relative frequency of GO term commonalities between those partners. Single MSAs or pairs of protein families consisting of more than 15% unknown organisms are not included in final statistics of orthologous sequences. All aforementioned processes finally leave pairs of MSAs consisting of potentially interacting orthologous partners arranged in the same species order.

Pairs of MSAs (dimers) consisting of less than 50 sequences are removed from the analysis. The final numbers of sequences in pairs of MSAs (N) ranges between $\sim 50$ and $\sim 950$.

We describe the final MSAs by $N/L$ ratio. This ratio varies between ∼0.2 and ∼6 in our datasets.

While the preliminary datasets contain more than 280 dimers, the final remaining numbers of samples after refinement process (filtration criteria, restrictions on L and N, outlier removal and, so forth) are 166 dimers in total, 83 in each dataset. Lists of positive and negative pairs of proteins are respectively available in Additional file 2: Table S1 and Table S2.

Only exposed residues on a protein surface potentially interact with their surface accessible partners on other proteins to form a heterodimer. Thus, binding interfaces of interacting pairs exist on surface accessible sites of each protein [53]. Accordingly, we entered additional information about Accessible surface area (ASA) into our MSA datasets for further covariation analysis. We hypothesized ten ASA thresholds whereby extracted ten pairs of Exposed-Residue MSAs (ER-MSA) from each preliminary constructed couple of MSAs. For that purpose, first an ASA is assigned to each residue position in primary sequence of each protein by the use of NetsurfP version 1.1 [63] and RaptorX property prediction webservers [64]. For ASA assignment to each residue component of protein molecules, those webservers only rely on protein sequences instead of having either known or unknown structures. ASA values for NetsurfP and RaptorX webservers are averaged for each amino acid position and a final ASA is reported for each residue column of the protein family. Ten ASA threshold of 0.0, 0.05, 0.15, 0.2, 0.25, 0.35, 0.45, 0.55, 0.65 and 0.75 are presumed. For each ASA threshold, amino acids exceeding the cut-off are postulated as exposed residues and thereby assumed as a part of hypothetical interacting site while residues falling behind the threshold are presumed as buried ones.

We constructed final pairs of MSAs for each of ten ASA thresholds which only consist of exposed residue columns (ER-MSAs). Thus, ER-MSAs contain all columns excluding those bound up with buried amino acids.

It should be noted, that in MI analysis, we do not concatenate MSAs of two interacting proteins. Since CCMpred was implemented using the package [37] which demands single paired MSAs, we concatenated ER-MSAs for this specific case.

Both final concatenated and non-concatenated ER-MSA datasets are available in Additional file 5 [65].

### Calculation and unification of whole inter-protein residue coevolution

Covariation between all residue columns of each pair of ER-MSAs is calculated using CCMpred [37] and three MI-based methods, i.e., MI [34], MIp [54] and Context Likelihood of Relatedness (CLR) [66]. Buried amino acids are indeed excluded and only coevolution between columns of surface accessible residues is calculated on pairs of MSAs in different ASA thresholds.

*MI*:

We calculate raw MI according to Martin et al. [34] through the following formulas:

1 $$MI\left( {x,y} \right) = H\left( x \right) + H\left( y \right) - H\left( {x,y} \right)$$

2 $$H\left( x \right) = - \sum\limits_{i = 1}^{21} f \left( {x_{i} } \right)\log_{21} f\left( {x_{i} } \right)$$

3 $$H\left( {x,y} \right) = - \mathop \sum \limits_{i = 1}^{21} \mathop \sum \limits_{j = 1}^{21} f\left( {x_{i} ,y_{j} } \right)\log_{21} f\left( {x_{i} ,y_{j} } \right)$$

where $H\left( x \right)$ and $H\left( {x,y} \right)$ are marginal and joint entropy, respectively. $f\left( {x_{i} } \right)$ is the relative frequency of residue $x$ at column $i$ in first MSA, $f\left( {y_{j} } \right)$ is the relative frequency of residue $y$ at column $j$ in the second MSA and $f\left( {x_{i} ,y_{j} } \right)$ is the co-occurrence frequency of residue pair $xy$ at column $ij$ between a pair of MSAs. Since there are 21 characters for proteins (20 amino acids and one extra character for gap), we apply logarithm of the base 21 [34].

We finally normalize MI with joint entropy to obtain standardized entropy effect on MI [67]:

4 $$MI_{normalized} = \frac{{MI\left( {x,y} \right)}}{{H\left( {x,y} \right)}}$$

To deal with gaps, columns with $\ge 80\%$ gaps are assumed to have zero MI.

*CLR*:

We compute CLR according to Faith et al. [66]. $Z_{i}$ is calculated as follows:

5 $$Z_{i} = \max \left( {0,\frac{{I\left( {x_{i} ,x_{j} } \right) - \mu_{i} }}{{\sigma_{i} }}} \right)$$

where $\mu_{i}$ and $\sigma_{i}$ are mean and standard deviation over column $i$ respectively. $\mu_{j}$ and $\sigma_{j}$ are also measured the same as $\mu_{i}$ and $\sigma_{i}$, but over row $j$ to calculate $Z_{j}$. $I\left( {x_{i} ,x_{j} } \right)$ is the element of normalized MI matrix at column $i$ and row $j$.

Final form of CLR at column $i$ and row $j$ is measured using the following formula:

6 $$f\left( {Z_{i } ,Z_{j} } \right) = \sqrt {Z_{i}^{2} + Z_{j}^{2} }$$

*MIp*:

According to Dunn et al. [54] $MIp\left( {a, b} \right)$ is calculated by the following formula:

7 $$MIp\left( {a,b} \right) = MI\left( {a,b} \right) - APC\left( {a,b} \right)$$

APC is defined as follows [54]:

8 $$APC\left( {a,b} \right) = \frac{{MI\left( {a,\overline{x}} \right)MI\left( {b,\overline{y}} \right)}}{{\overline{MI} }}$$

where $MI\left( {a,\overline{x}} \right)$ and $MI\left( {b,\overline{y}} \right)$ are denoted as the mean MI values of column $a$ over $x = 1$ to $x = n$, and the mean MI values of row $b$ over $y = 1$ to $y = m$, respectively. $\overline{MI}$ is the mean MI value of whole matrix.

In the original papers for MIp [54] and CLR [66], those covariations are calculated for single MSAs and thereby square matrices are constructed and the above mentioned mean values are calculated over off-diagonal entries. In the present study, we have not concatenated pairs of MSAs for assessing MI-based approaches which results in the formation of rectangular covariation matrices with no exclusions on diagonal entries for calculation of mean values.

*CCMpred*:

CCMpred is an optimized and fast Pseudo-Likelihood Maximization-based (PLM-based) procedure, disentangling direct residue couplings from indirect ones [37]. A package is available for implementing the method which takes single MSAs as input. Therefore, we concatenate two MSAs for each dimer in order to implement this method. The final couplings between sequences is trimmed out of the whole available inter and intra-couplings.

*Unification of covariation matrices*:

We obtain "Frobenius norm" of each inter-protein residue interaction matrix to summarize and unify whole elements of each matrix into a single meaningful quantity. To calculate Frobenius norm [68] of each residue coevolution $m \times n$ matrix which belongs to an interacting pair of proteins, we apply the following expression in which the $a_{ij}$ ‘s are matrix entries:

9 $$A_{F} = \left( {\mathop \sum \limits_{i. j = 1}^{n} \left| {a_{ij} } \right|^{2} } \right)^{\frac{1}{2}}$$

### Discrimination of positive and negative heterodimers

In order to make discrimination between pairs of positive and negative (control) datasets, we implement several SVM classifications. The input features of SVM models are the Frobenius norm values of covariation matrices (Fig. 4b). SVM takes single norm features of positive and negative heterodimers in each ASA threshold for building single feature model (Fig. 1a). Multiple groups of norm values for a set of ASA thresholds are also fed into SVM classifiers as multiple input features (Fig. 1b).

The radial kernel function [69] is applied in all of the SVM models as:

10 $$k\left( {a_{i} ,a_{j} } \right) = {\exp}\left( {\frac{{\left( { - 1} \right)a_{i} - a_{j}^{2} }}{{2\sigma^{2} }}} \right)$$

where $\sigma$ is a free parameter. $a_{i}$ and $a_{j}$ also indicate norm values of proteins $i$ and $j$ of each dimer, respectively.

We hold out 30% of whole datasets as a test set for the final evaluation and apply tenfold cross-validation on the remaining 70%. The cross validation subset of our data is randomly split into nine equal parts. To guarantee the thorough independence of training procedure from validation data, SVMs are trained on nine parts of the data and validated on the remaining one. The SVMs are performed using "e1071" R package. The model parameters are tuned by rotating the training and validation data for ten times, using "tune" function. The final tuned models are tested on the previously set aside test set.

Prediction performance are evaluated using a set of evaluation metrics, i.e., Accuracy, Sensitivity, Specificity, Precision, False Positive Rate (FPR), Negative Predictive Value (NPV), False Negative Rate (FNR) and Bookmaker Informedness (BM) defined as follows:

11 $$Accuracy = \frac{TP + TN}{{TP + TN + FP + FN}}$$

12 $${\text{Sensitivity}} = \user2{ }\frac{{{\text{TP}}}}{{{\text{TP}} + {\text{FN}}}}$$

13 $$Specificity = \user2{ }\frac{{{\text{TN}}}}{{{\text{TN}} + {\text{FP}}}}$$

14 $$Precision = \user2{ }\frac{{{\text{TP}}}}{{{\text{TP}} + {\text{FP}}}}$$

15 $$FPR = \user2{ }\frac{{{\text{FP}}}}{{{\text{FP}} + {\text{TN}}}}$$

16 $$NPV = \user2{ }\frac{{{\text{TN}}}}{{{\text{TN}} + {\text{FN}}}}$$

17 $$FNR = \user2{ }\frac{{{\text{FN}}}}{{{\text{FN}} + {\text{TP}}}}$$

18 $$BM = Sensitivity + Specificity - 1$$

where TP, TN, FP, and FN denote true positive, true negative, false positive, and false negative, respectively.

A non-routine evaluation metric, BM, is measured to assess the trade-off between sensitivity and specificity.

## Supplementary Information

**Additional file 1.** Norm values of whole inter-protein residue interactions in specific ASA thresholds.

**Additional file 2.** Supplemental dataset construction, figures and tables.

**Additional file 3.** Prediction evaluation results for SVM models developed by single norm features for separate ASA thresholds in comparison with all norm features for all ASA thresholds on CCMpred and MI-based methods. Accuracy, sensitivity, specificity, precision, NPV, FPR, FNR and Informedness results of CCMpred, MI, MIp and CLR methods are compared in groups of tables for single norm features in separate ASA thresholds and all norm features in all ASA groups.

**Additional file 4.** SVM prediction evaluation results for multiple feature sets in groups of ASA thresholds. Accuracy, sensitivity, specificity, precision, NPV, FPR, FNR and Informedness results of CCMpred, MI, MIp and CLR methods are compared in groups of tables..

**Additional file 5.** Final ER-MSAs at different ASA thresholds. Available on Mendeley data (http://dx.doi.org/10.17632/9bk2r55286.1).

## Data Availability

Evaluation tables, prediction results, norms of each dimer for all ASA thresholds, supplementary figures and the lists of PDB interacting chains used in this experiment are included within the additional files. The ER-MSA datasets generated at different ASA thresholds are available on Mendeley Data (http://dx.doi.org/10.17632/9bk2r55286.1).

## Abbreviations

ASA: Accessible surface area
BM: Bookmaker informedness
CLR: Context likelihood of relatedness
CCMpred: Contacts from correlated mutations predicted
DCA: Direct coupling analysis
ER-MSA: Exposed-residue multiple sequences alignment
FNR: False negative rate
FPR: False positive rate
HOE: Homologous over-extension errors
MI: Mutual Information
MIp: Mutual information product
MSA: Multiple sequence alignment
NPV: Negative predictive value
PDB: Protein Data Bank
PPI: Protein–protein interaction
PPV: Positive predictive value
SVM: Support vector machine
