# Supplementary material for: Inter-protein residue covariation information unravels physically interacting protein dimers
Source: BMC Bioinformatics. 2020 Dec 17;21:584. doi: 10.1186/s12859-020-03930-7 (PMC7745481; doi:10.1186/s12859-020-03930-7)
Supplement: Supplementary file 2 — Additional file 2. Supplemental dataset construction, figures and tables. [file 12859_2020_3930_MOESM2_ESM.docx]

Supplementary Information

**Inter-protein residue covariation information unravels physically interacting protein dimers**

Sara Salmanian^1^, Hamid Pezeshk^2,3,^ *, Mehdi Sadeghi^4^

^1^ Department of Bioinformatics, Institute of Biochemistry and Biophysics, University of Tehran, Tehran, Iran

^2^ School of Mathematics, Statistics and Computer Science, College of Science, University of Tehran, Tehran, Iran (currently visiting Department of Mathematics and Statistics, Concordia University, Montreal, Canada)

^3^ School of Biological Sciences, Institute for Research in Fundamental Sciences, Tehran, Iran

^4^ National Institute of Genetic Engineering and Biotechnology, Tehran, Iran

*Corresponding Author: [pezeshk@ut.ac.ir](mailto:pezeshk@ut.ac.ir)

**Contents:**

1. [**Dataset Construction**](#dataset) …………………………………………………………….... [**2**](#dataset)
   1. [**Selection of Orthologous Sequences and Removing Paralogs**](#ortholog) ………........... [**2**](#ortholog)
   2. [**Filtration Criteria**](#filtration) …………………………………………………………….. [**3**](#filtration)
   3. [**Algorithm for Detecting Outliers**](#outlier_detection) ……………………………………………. [**4**](#outlier_detection)
2. [**Figures**](#Figures) ………………………………………………………………………………[**5**](#Figures)
3. [**Tables**](#Tables3) ………………………………………………………………………………[**13**](#Tables3)
4. **Dataset Construction**
   1. **Selection of Orthologous Sequences and Removing Paralogs**

We applied four steps to select orthologous sequences and remove paralogous proteins from the final protein families:

- To find homologous sequences, we applied PSI-search [1] which reduces Homologous Over-Extension Errors (HOE) whereby both less redundant and most similar sequences to query are found. Therefore, after employing this step only a single or a handful of homologous proteins are detected for each species.
- In each iterative step of PSI-search, we applied a set of filtration criteria to sieve a group of eligible homologous sequences. Those criteria are stringently set up such that solely most similar and functionally relevant sequences (with identical GO terms) to query are selected. Due to the exclusion of less similar and functionally different homologous proteins by those filtrations, selection of paralogous proteins is less likely at this step on account of "orthologue conjecture" [2]. Hence, the majority of homologous sequences are deemed to be orthologues at this point.
- Although it is assumed that most of paralogous sequences are eliminated at two aforementioned steps, but there are still a few species consisting of more than a single homologous sequence. It could be due to the fact that some paralogs may retain their function and resemble query yet. At this point, one of those sequences were randomly selected for each species.
- We conceptualized that even if the randomly selected sequence is a paralogous one, it would be considerably different from other selected members of the protein family on account of gene duplication or gene loss and thereby exert huge gaps within the MSA of that family (outlier sequences as it would be noted later). Accordingly, it is presumed that the final elimination of outlier sequences and their corresponding counterparts, would result in removing incorrectly selected paralogous sequences.

As earlier mentioned, sequences were filtered at lower identities by applying filtration criteria but there was also a corpus of redundant and highly identical sequences. Redundant sequences were simply eliminated but highly identical sequences could make the final MSAs inappropriate for covariation analysis. Since most of detected homologous sequences were prokaryotic and proteins of different species of a genus are highly identical in prokaryotes and even in some cases there are multispecies sequences, among a set of species of a genus we randomly selected only a single one to build higher distinctive MSAs in later steps (Fig S2).

- 1. **Filtration Criteria**

The following script summarizes filtration criteria in more details:

$$overlap=\frac{overlap length}{Hit length}$$

$$if \left( bit score \geq0.5 \times quety length \right) and \left( 0.8 \times query length\leq Hit length \leq1.2 \times query length \right) and \left( identity \geq25\% \right) and \left( 0.9 \leq overlap \leq1.1 \right) and Evalue \leq0.0001:$$

$$select the Hit$$

$$elif \left( bit score \geq0.5 \times quety length \right) and \left( 0.8 \times query length\leq Hit length \leq1.2 \times query length \right) and \left( 30\%\leq identity\leq35\% \right) and \left( 0.85 \leq overlap \leq1.1 \right) and Evalue \leq0.0001:$$

$$select the Hit$$

$$elif \left( bit score \geq0.5 \times quety length \right) and \left( 0.8 \times query length\leq Hit length \leq1.2 \times query length \right) and \left( 35\%\leq identity\leq40\% \right) and \left( 0.8 \leq overlap \leq1.1 \right) and Evalue \leq0.0001:$$

$$select the Hit$$

$$elif \left( bit score \geq0.5 \times quety length \right) and \left( 0.8 \times query length\leq Hit length \leq1.2 \times query length \right) and \left( identity \geq40\% \right) and \left( 0.7 \leq overlap \leq1.1 \right) and Evalue \leq0.0001:$$

$$select the Hit$$

$$elif \left( bit score \geq0.5 \times quety length \right) and \left( 0.78 \times query length\leq Hit length \leq1.22 \times query length \right) and \left( identity \geq40\% \right) and \left( 0.9 \leq overlap \leq1.1 \right) and Evalue \leq0.0001:$$

$$select the Hit$$

$$elif \left( bit score \geq0.45 \times quety length \right) and \left( 0.8 \times query length\leq Hit length \leq1.2 \times query length \right) and \left( identity \geq40\% \right) and \left( 0.9 \leq overlap \leq1.1 \right) and Evalue \leq0.0001:$$

$$select the Hit$$

$$else:$$

$$discard the Hit$$

- 1. **Algorithm for Detecting Outliers**

As earlier mentioned in section 1.1, most outliers are probably those paralogous sequences with similar function to other members of the protein family which exert huge gaps within MSAs.

In each MSA, those sequences are probably proteins suspicious to be the product of genes undergone gene duplication or gene loss thereby hypothesized as probable paralogs.

We designed a simple algorithm for eliminating outlier sequences. If gap percentage in each MSA column is more than 70% or less than 30%, that column is remarked as "Determinative column". For each single homologous sequence, if the residue in each "Determinative column" is different from other 70% residues residing on homologous sequences in that column, we add a count to a scale called "Determinative-Column-Count-Difference" (DCCD). Finally, a metric called "Homologous Removal Criterion" (HRC) is calculated for each sequence which is the percentage of DCCD over whole MSA length:

$$HRC= \frac{DCCD}{MSA length} \times100 (1)$$

If HRC of a sequence exceeds a user-prespecified threshold (2% in our dataset), that sequence is marked as an outlier and removed from MSA (Fig. S3).

1. **Figures**


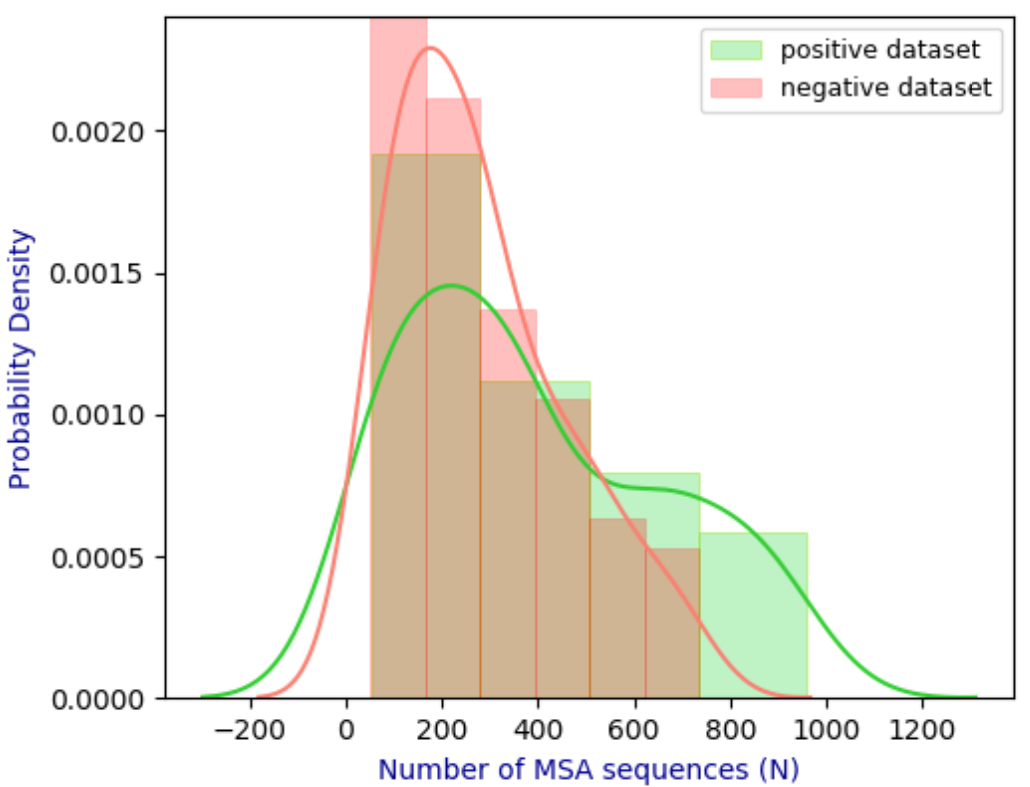


**Fig. S1-** Distribution of the numbers of sequences in MSA datasets


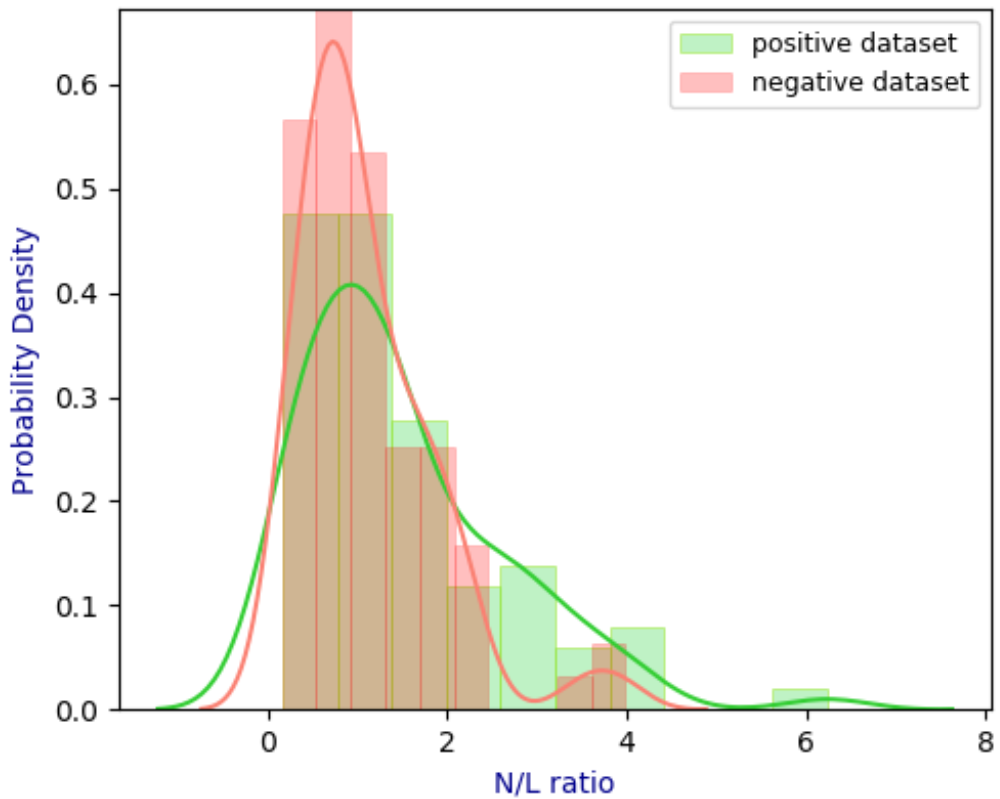


**Fig. S2-** Distribution of $N/L$ratio in MSA datasets


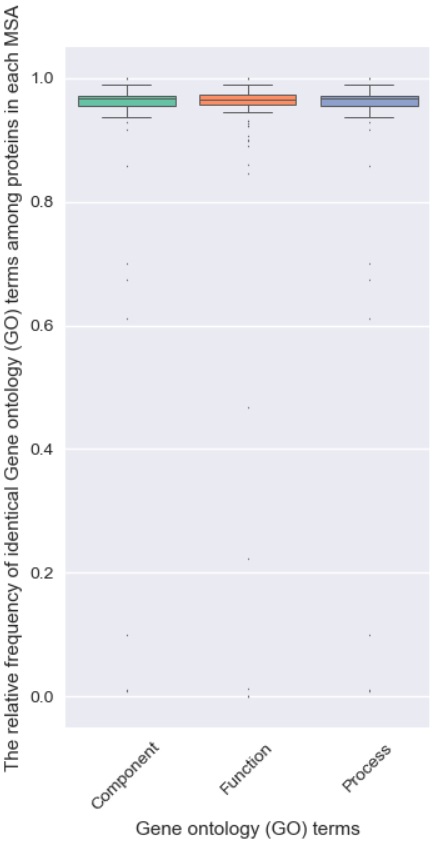


**Fig. S3-** Distribution of GO term identities between sequences of protein families and query proteins


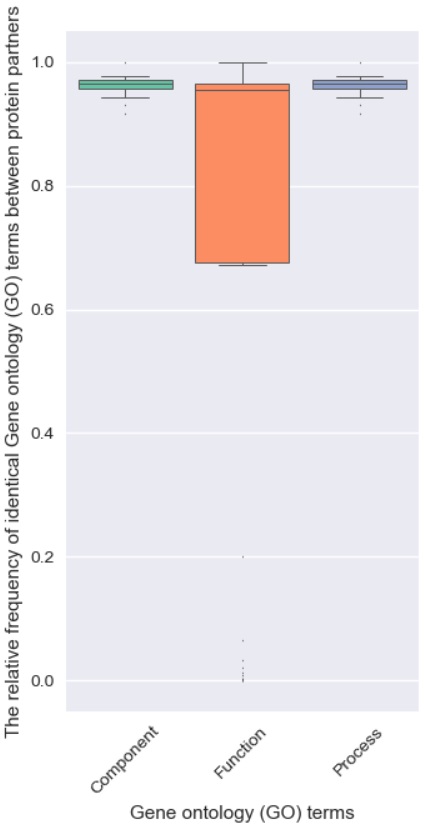


**Fig. S4-** Distribution of GO term identities between protein partners in MSA dataset


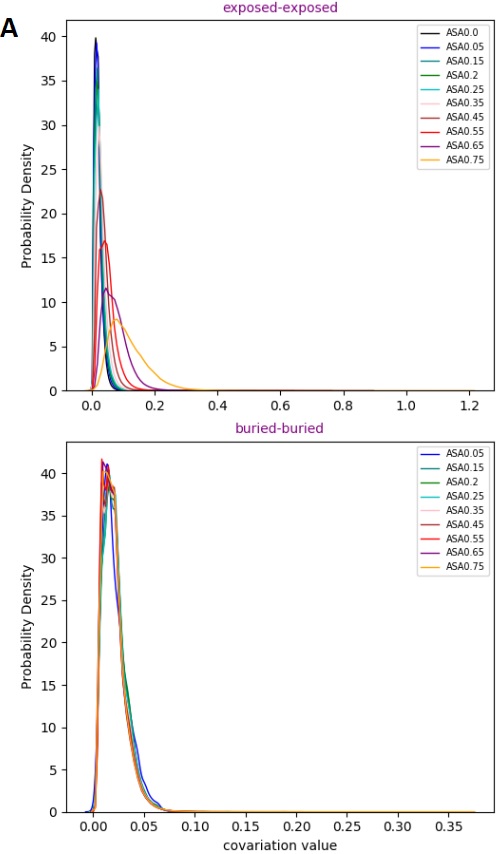

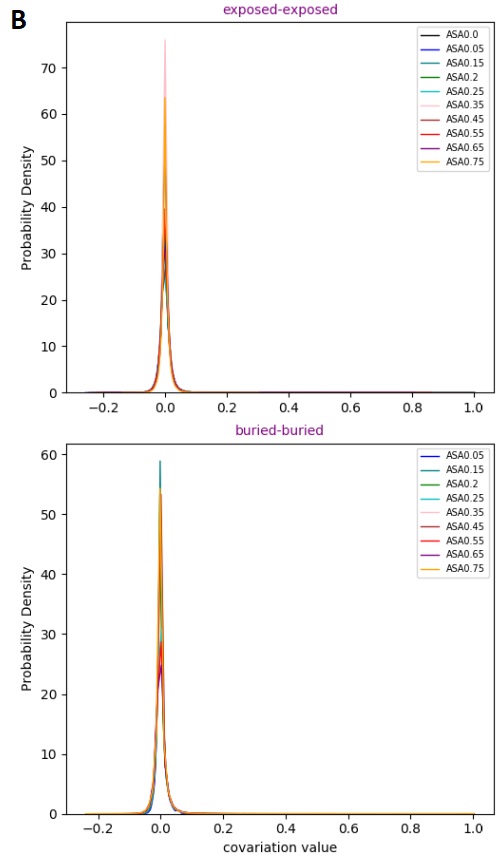
**
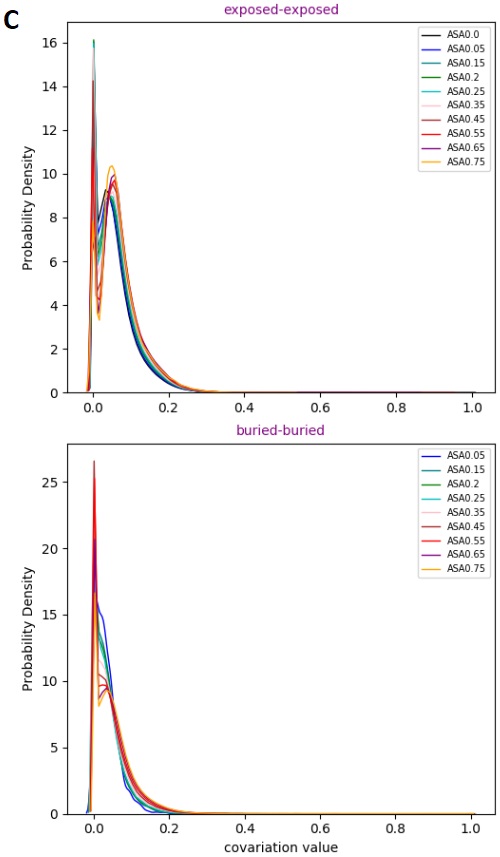

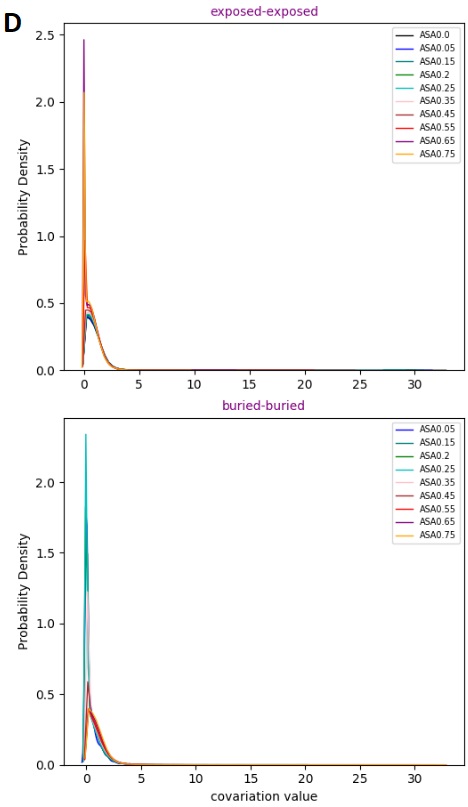
**

**Fig. S5-** Covariation value distributions of buried and exposed residue pairs in CCMpred and three MI-based approaches for different ASA thresholds. **A**, **B**, **C** and **D** illustrate distributions of covariation values of buried and exposed pairs in separate ASA thresholds in CCMpred, MIp, MI and CLR methods, respectively. As shown by this figure, there is an apparent distinction among different ASA values between buried and exposed residues in CCMpred approach.


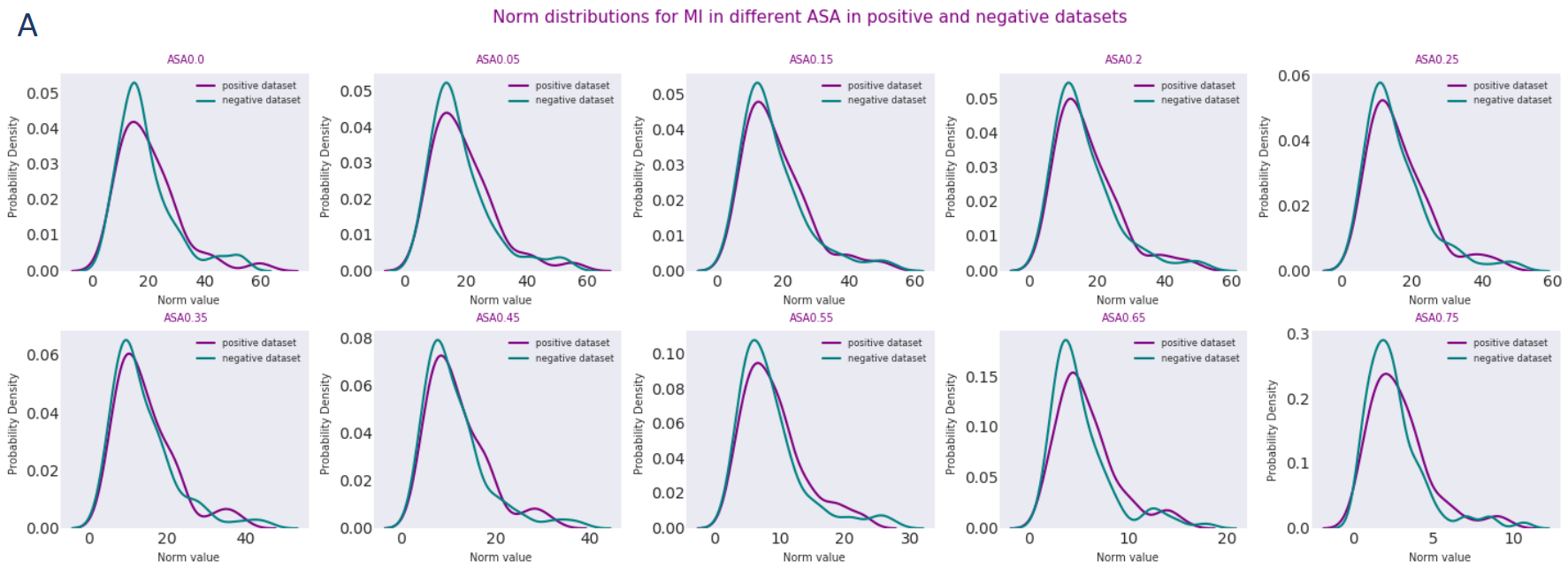


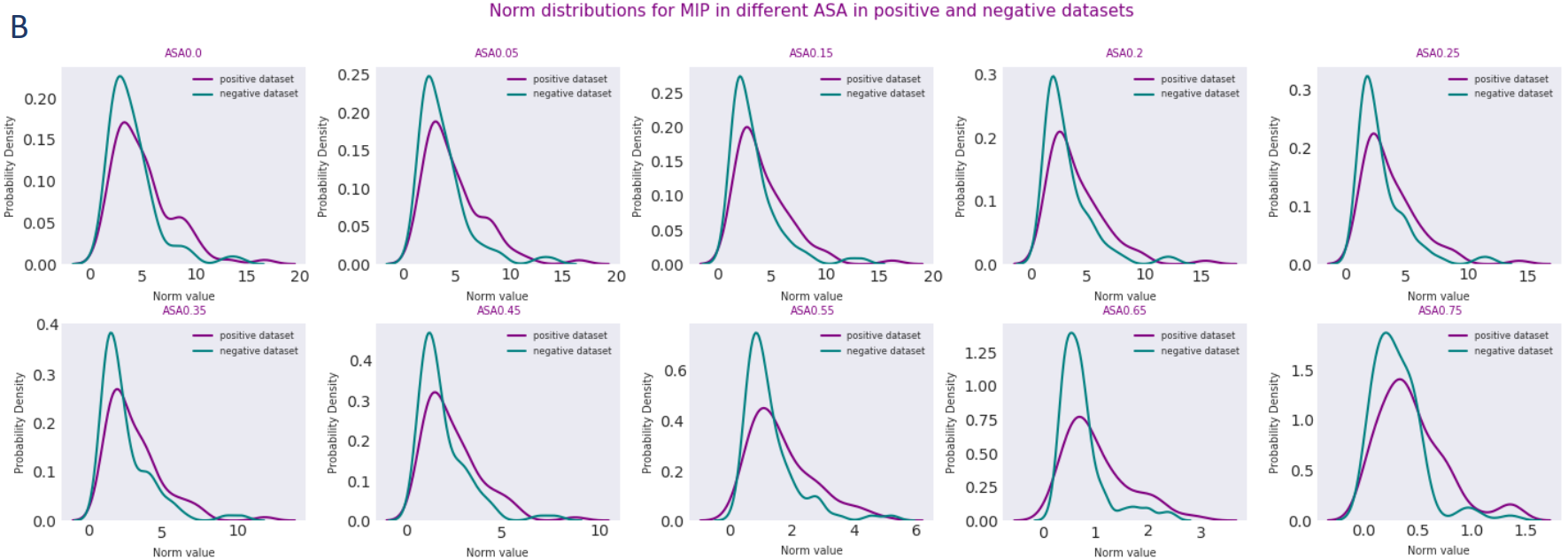


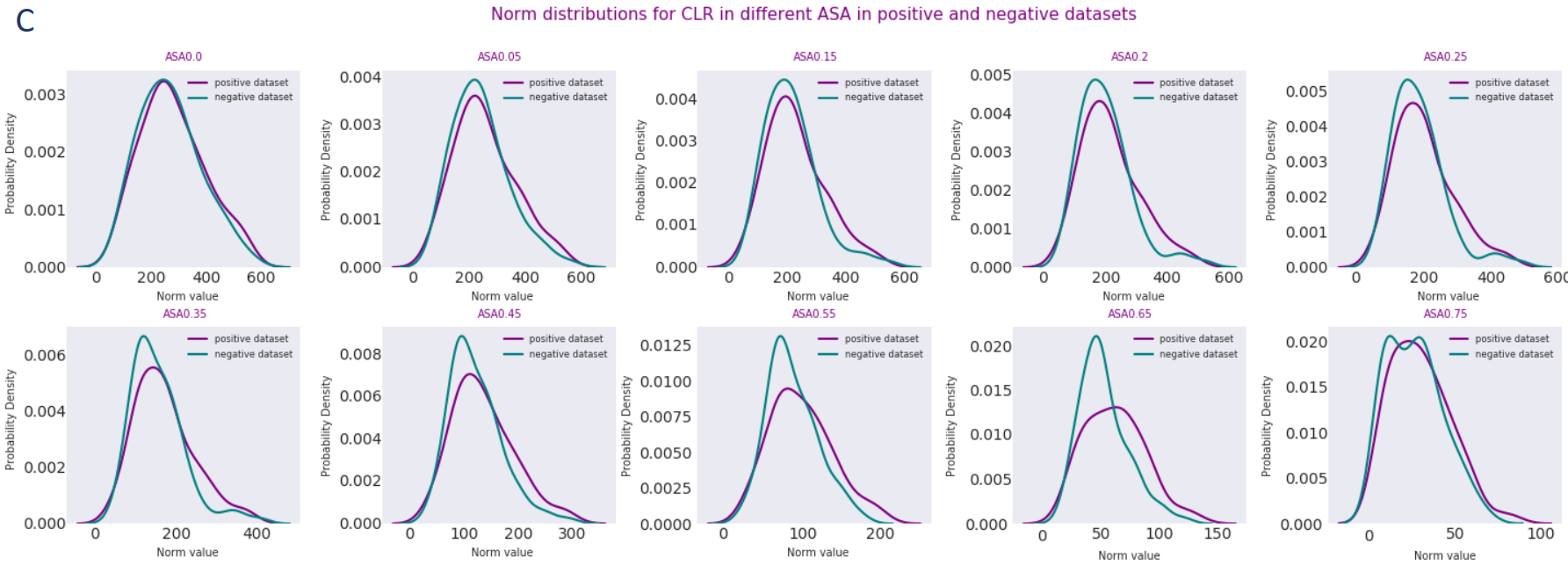


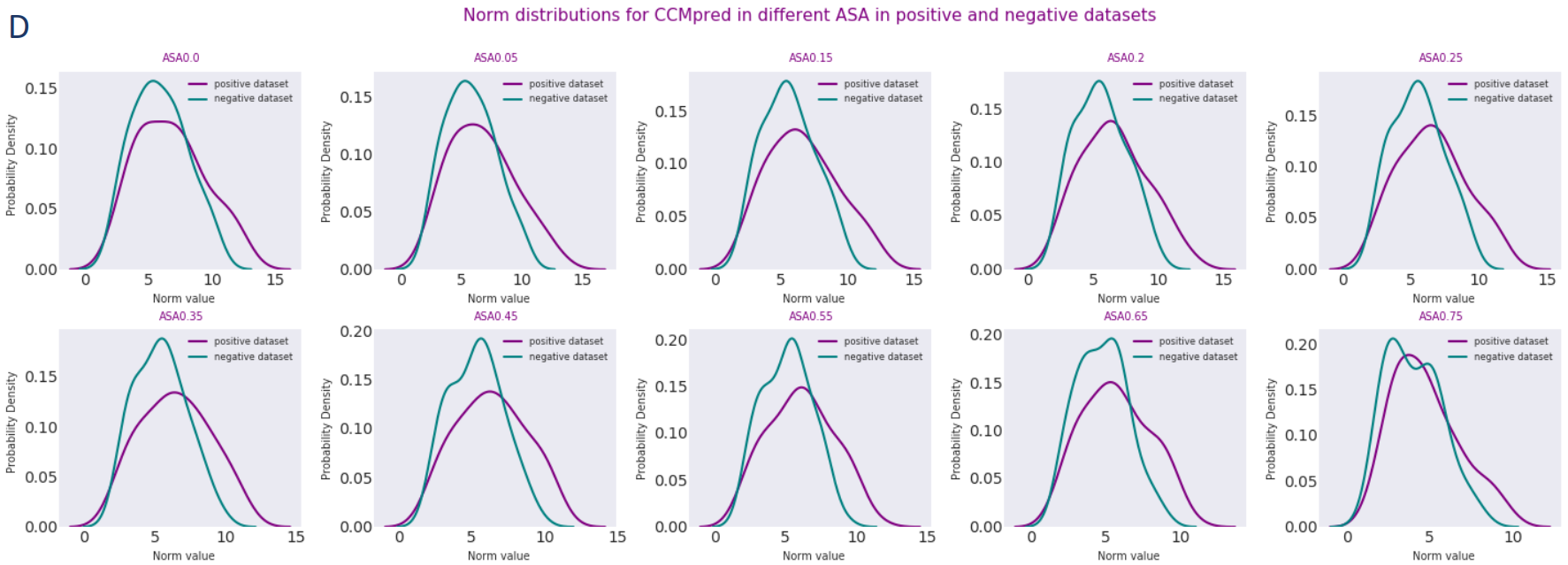


**Fig. S6 -** Distributions of single norm features in separate ASA thresholds in both positive and negative datasets in CCMpred and three distinct MI-based methods. **A**, **B**, **C** and **D** illustrate distributions of norm values in single separate ASA thresholds in MI, MIp, CLR and CCMpred methods, respectively.

B

A

**
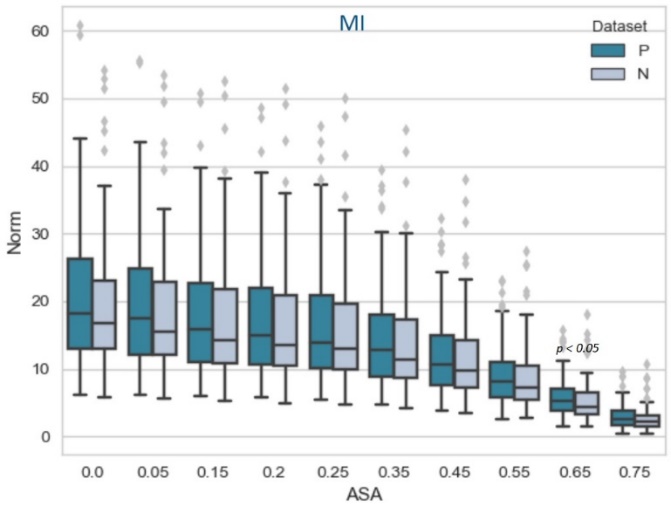

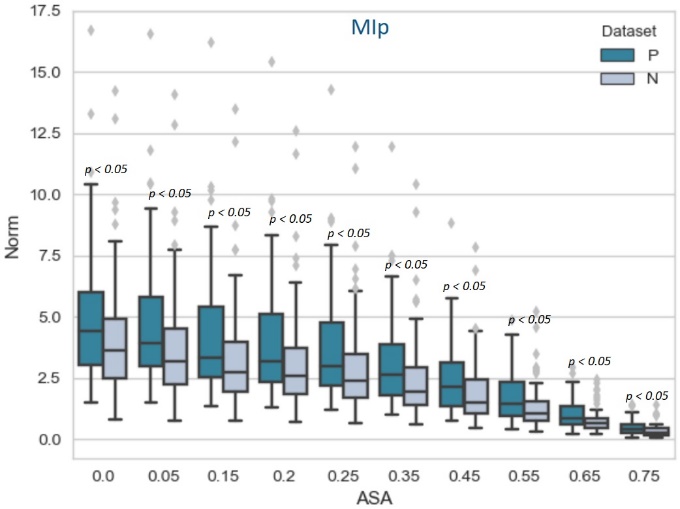
**

D

C

**
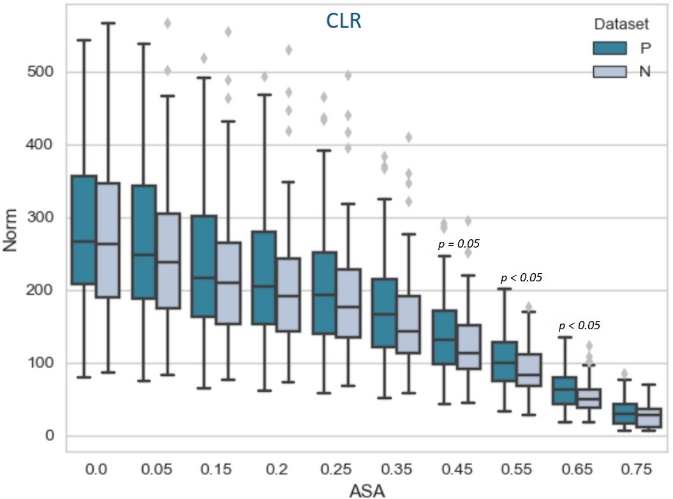

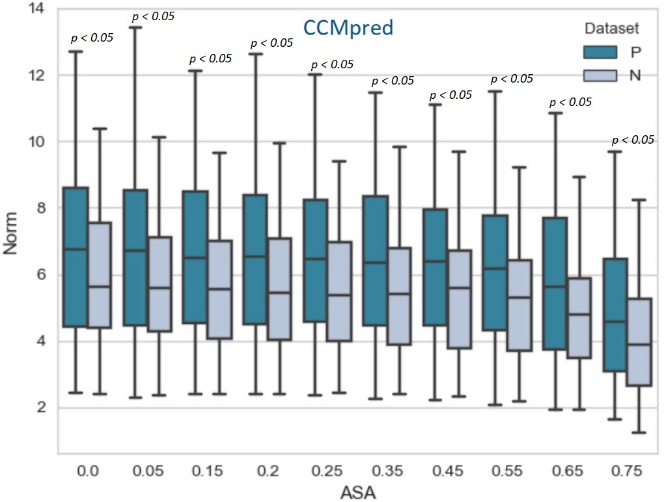
**

**Fig. S7 -** Box plots for single norm features in separate ASA thresholds in both positive and negative datasets in CCMpred and three distinct MI-based methods. **A**, **B**, **C** and **D** illustrate box plots of norm values in single separate ASA thresholds in MI, MIp, CLR and CCMpred methods, respectively. A Mann-Whitney U test was performed with a significance level of 5% or lower. The p-value of significantly different pairs are indicated on the graphs (p < 0.05). The difference between averages of norm values of positive and negative datasets are significantly different in all ASA thresholds in CCMpred and MIp approaches, and only different at ASA= 0.65 in MI method and ASA= 0.45 to ASA= 0.65 in CLR approach.


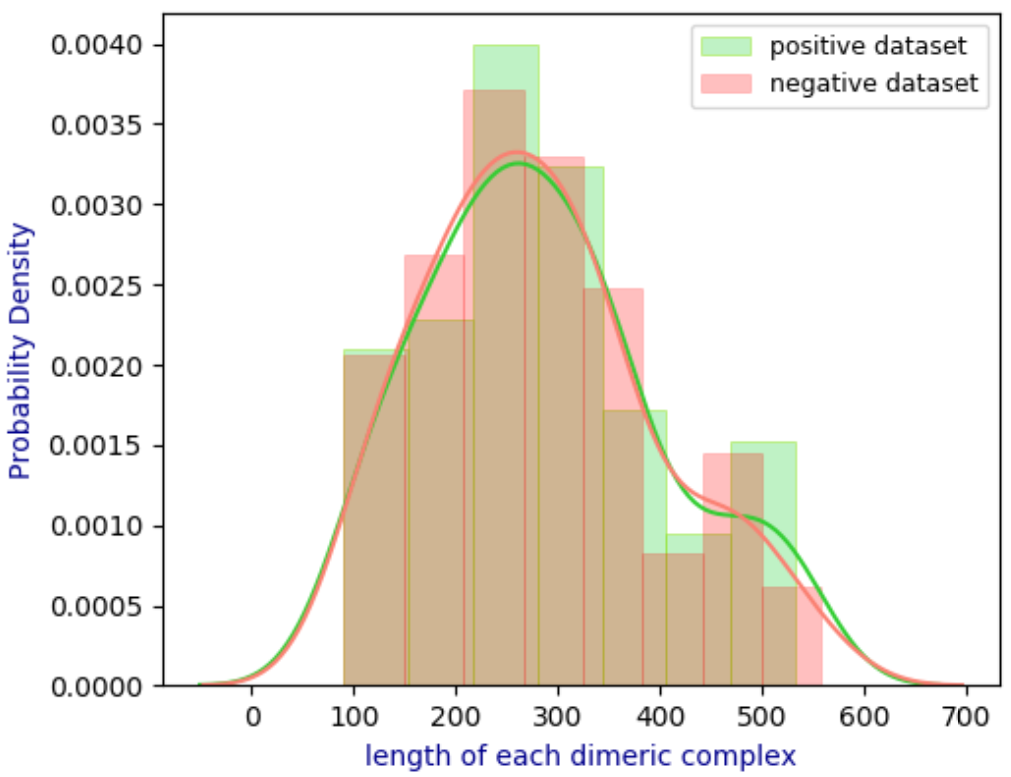


**Fig. S8-** Distribution of the lengths of heterodimers in positive and negative datasets


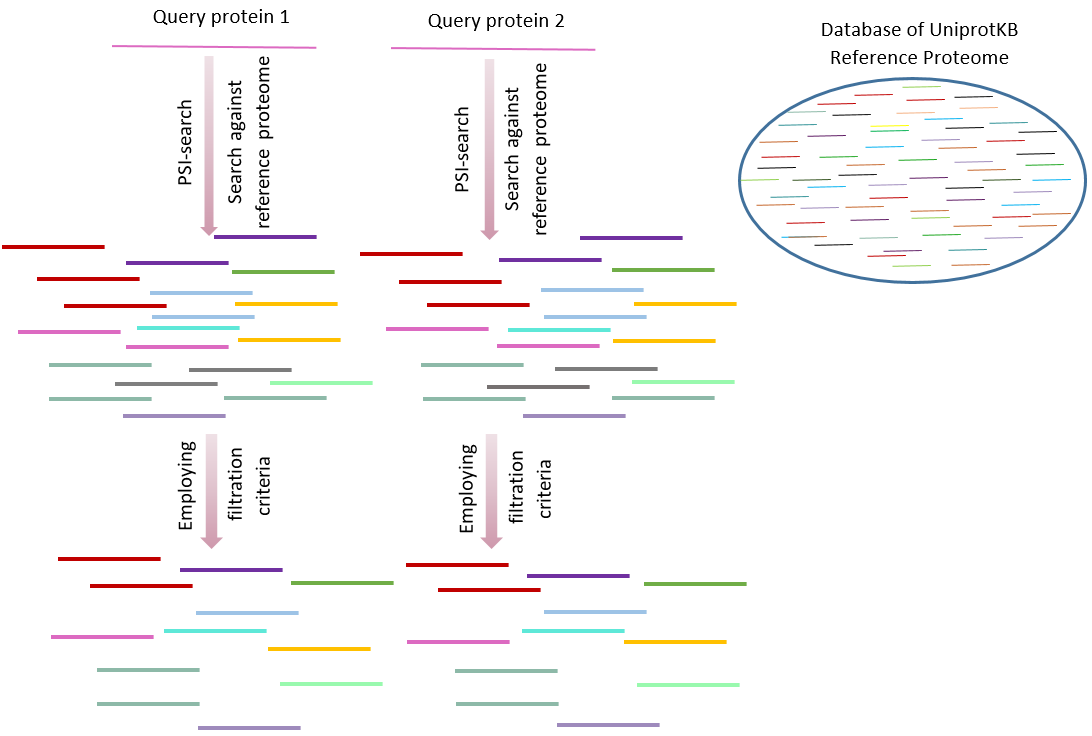


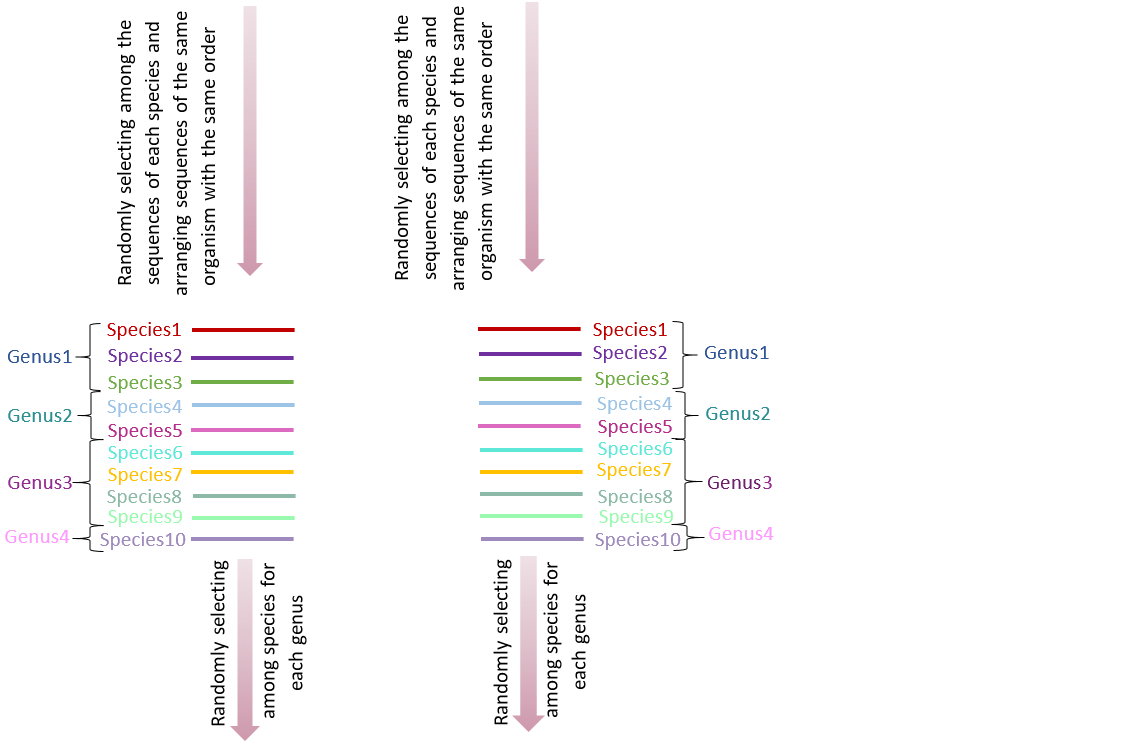


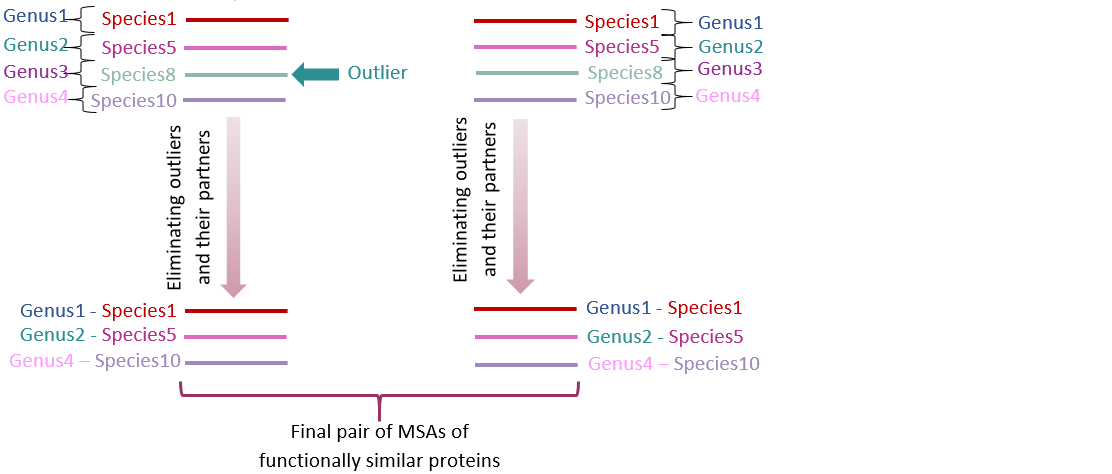


**Fig. S9-** Detailed pipeline of orthologue selection. There are four steps for removing paralogous and selecting orthologous sequences: i) PSI-search similarity search by reducing HOE; ii) Applying filtration criteria; iii) Randomly selecting a single protein among the sequences of each species; iiii) Removing outlier sequences from MSAs


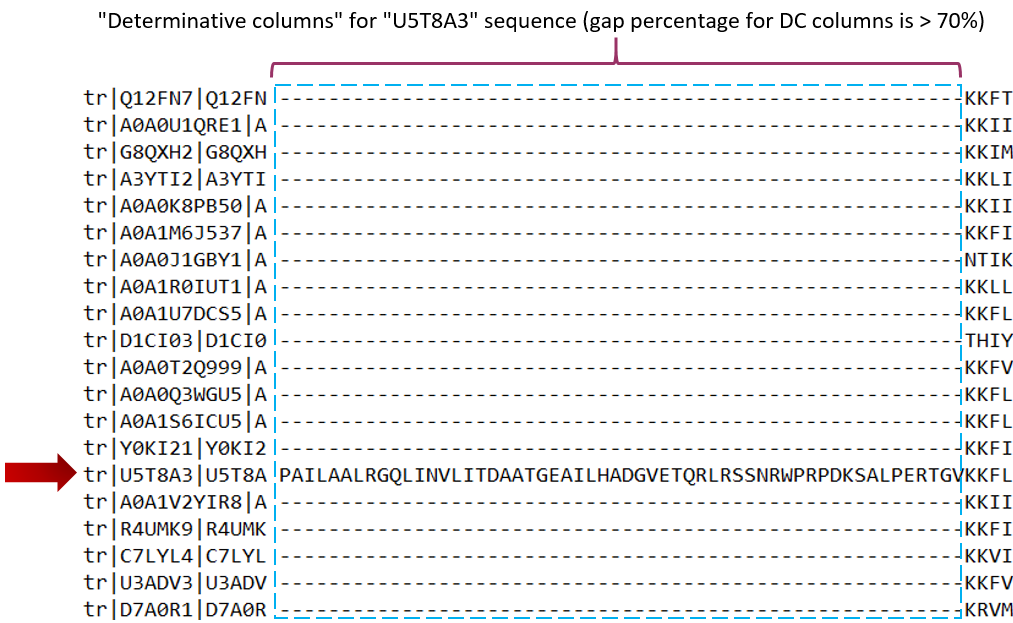


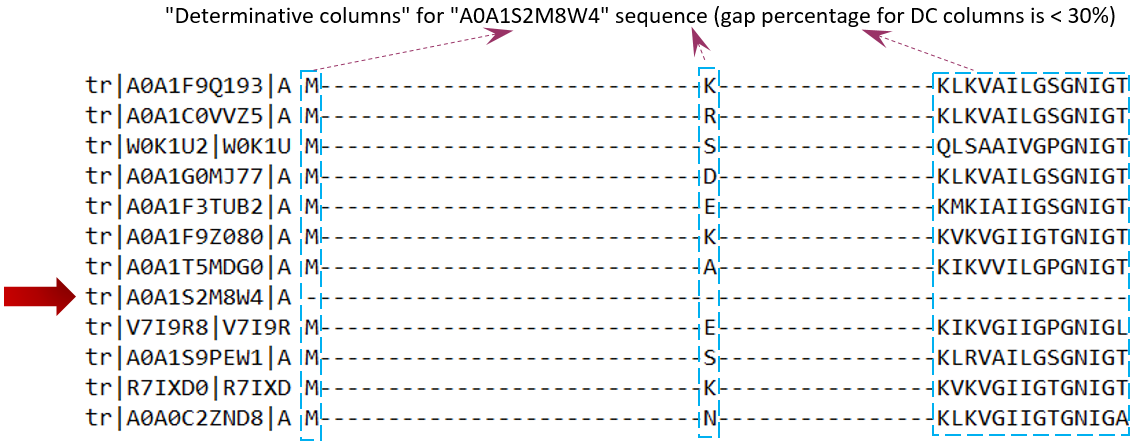


**Fig. S10-** Examples of outliers. Outliers are those sequences highly different from other sequences of a protein family in "Determinative columns". Thick red arrows indicate outlier sequences. DC: Determinative Column

1. **Tables**

**Table S1- List of positive dimers**

|  | |  | | Positive Heterodimeric Complexes | | | |  |  |
| --- | --- | --- | --- | --- | --- | --- | --- | --- | --- |
| 1q16-B-C | | 3pnl-B-A | | 4hea-F-G | 1lzw-A-B | 1wdk-A-C | | | |
| 4hea-J-N | | 2a7u-A-B | | 1rm6-A-C | 1tyg-B-A | 1l7v-A-C | | | |
| 2b3t-B-A | | 1g4a-E-C | | 2hqs-A-H | 3ip4-B-C | 1w85-A-B | | | |
| 3oaa-D-H | | 3a8i-E-A | | 1kf6-A-C | 2y69-A-C | 2d1p-C-B | | | |
| 1zun-B-A | | 1kqf-B-C | | 1nek-B-D | 4hea-L-M | 3oaa-A-G | | | |
| 2r6g-G-A | | 4hea-K-N | | 1ixr-C-B | 2qi9-A-F | 3r9j-C-A | | | |
| 2wiu-B-A | | 1acm-A-B | | 3abo-A-B | 3mml-A-B | 2r6g-F-E | | | |
| 2ns1-B-A | | 1fft-A-B | | 4hea-M-N | 3teh-A-B | 4hea-H-J | | | |
| 1fft-A-C | | 2r6g-E-G | | 4hea-A-H | 4hea-L-N | 3vti-C-A | | | |
| 1fm0-D-E | | 1kf6-C-B | | 1kf6-D-B | 1nvm-B-A | 2r6g-F-B | | | |
| 1aon-A-O | | 2y69-A-B | | 1nek-B-C | 3oaa-A-D | 2nu9-A-B | | | |
| 3oaa-A-H | | 1rm6-A-B | | 1efp-A-B | 3rrl-A-B | 1ep3-A-B | | | |
| 4hea-A-J | | 2d1p-C-A | | 4hea-J-K | 2wdq-C-D | 1nek-B-A | | | |
| 3oaa-D-G | | 1kf6-A-B | | 4fhr-A-B | 4eb7-C-A | 3oaa-G-H | | | |
| 1rm6-B-C | | 3g5o-A-B | | 1a0o-B-A | 3n39-C-B | 1kmi-Z-Y | | | |
| 1kf6-C-D | | 1q16-A-C | | 2avu-A-E | 2zu0-C-A | 1qop-A-B | | | |
| 4hea-A-K | | 3dhw-C-A | | 3ip4-A-C |  |  | | | |
|  | |  | |  |  |  | | | |

**Table S2- List of negative dimers**

|  |  | | Negative Heterodimeric Complexes | | | |  |  |
| --- | --- | --- | --- | --- | --- | --- | --- | --- |
| 1zun-A-2zu0-C | | 2ns1-B-4eb7-A | | 1efp-A-1qop-A | 1efp-A-3rrl-B | 4hea-N-3g5o-B | | |
| 2nu9-A-3ip4-B | | 3ip4-B-3mml-B | | 2zu0-C-4eb7-A | 1kf6-C-1aon-O | 4hea-M-2wdq-C | | |
| 1w85-B-1ep3-B | | 1qop-B-3rrl-A | | 1ep3-B-1qop-A | 1tyg-A-3mml-B | 1ep3-B-1rm6-C | | |
| 1qop-A-1rm6-A | | 1kf6-D-1aon-A | | 4hea-N-2y69-A | 4hea-K-2y69-A | 1efp-A-1qop-B | | |
| 4hea-M-3g5o-A | | 4hea-A-2wdq-C | | 1zun-A-2zu0-A | 1zun-A-4eb7-A | 1ep3-B-1qop-B | | |
| 4hea-L-2y69-A | | 3g5o-B-2y69-A | | 1kf6-B-1aon-O | 4hea-J-2wdq-C | 2ns1-A-4eb7-C | | |
| 4hea-L-2wdq-D | | 1efp-A-1rm6-A | | 1kf6-B-1aon-A | 4hea-M-2y69-B | 2ns1-B-4eb7-C | | |
| 1zun-B-4eb7-C | | 2ns1-B-2b3t-A | | 1ep3-A-3rrl-B | 4hea-H-2y69-B | 4hea-J-2wdq-D | | |
| 4hea-K-3g5o-B | | 1ep3-B-1rm6-A | | 1qop-A-3rrl-A | 4hea-J-3g5o-A | 3ip4-C-3mml-B | | |
| 1zun-B-4eb7-A | | 1kf6-D-1aon-O | | 1ep3-A-1qop-B | 1w85-B-1ep3-A | 2ns1-A-2b3t-A | | |
| 1w85-A-1ep3-B | | 2y69-A-2wdq-D | | 2zu0-C-3pnl-B | 4hea-L-2y69-B | 1efp-B-1rm6-A | | |
| 1fm0-D-1zun-A | | 4hea-A-2y69-B | | 1efp-B-1rm6-C | 4hea-K-2wdq-D | 4hea-H-2wdq-D | | |
| 1fm0-E-3pnl-B | | 1efp-A-1rm6-C | | 1ep3-A-1qop-A | 1tyg-A-3mml-A | 2zu0-C-4eb7-C | | |
| 3ip4-B-3mml-A | | 4hea-N-2wdq-C | | 1efp-B-1qop-B | 4hea-L-2wdq-C | 4hea-H-2y69-A | | |
| 1qop-B-1rm6-A | | 2zu0-A-3pnl-A | | 1zun-B-2zu0-A | 1ep3-A-1rm6-C | 4hea-M-2y69-A | | |

**References:**

1. Li W, McWilliam H, Goujon M, Cowley A, Lopez R, Pearson WR. PSI-Search: iterative HOE-reduced profile SSEARCH searching. Bioinformatics (Oxford, England). 2012;28(12):1650-1. doi: 10.1093/bioinformatics/bts240. PubMed PMID: PMC3371869.

2. Altenhoff AM, Studer RA, Robinson-Rechavi M, Dessimoz C. Resolving the Ortholog Conjecture: Orthologs Tend to Be Weakly, but Significantly, More Similar in Function than Paralogs. PLOS Computational Biology. 2012;8(5):e1002514. doi: 10.1371/journal.pcbi.1002514.
